# Antibacterial and antibiofilm effects of natural phenolic acids: Insights into structure-function relationships, extracellular pH, and morphological changes

**DOI:** 10.1128/spectrum.01336-26

**Published:** 2026-07-17

**Authors:** Ance Bārzdiņa, Renāte Teterovska, Daniela Paula Prudņikova, Renārs Broks, Krisjanis Smits, Ingus Skadiņš, Dagnija Loca, Aiva Plotniece, Arkadij Sobolev, Karlis Pajuste, Agnese Brangule, Dace Bandere

**Affiliations:** 1 Department of Applied Pharmacy, Riga Stradins University, Riga, Latvia; 2 Baltic Biomaterials Centre of Excellence, Headquarters at Riga Technical University, Riga, Latvia; 3 Department of Biology and Microbiology, Riga Stradins University, Riga, Latvia; 4 Institute of Biomaterials and Bioengineering, Faculty of Natural Sciences and Technology, Riga Technical University 87254, Riga, Latvia; 5 Latvian Institute of Organic Synthesis 187008, Riga, Latvia; 6 Department of Pharmacology and Pharmacotherapy, Riga Stradins University, Riga, Latvia; Universita degli Studi Roma Tre, Rome, Italy

**Keywords:** wound infection, biofilms, bacterial morphology, phenolic acids, hydroxycinnamic acids, hydroxybenzoic acids, ADMET

## Abstract

Wound infections are a challenging healthcare burden against which treatment is hindered by the presence of biofilms and the rise in antibacterial resistance. To date, the potential of natural phenolic acids as topical antibacterial agents is under-researched. The aim of this study was to investigate and compare the antibacterial and antibiofilm effects of 5 hydroxycinnamic acids (caffeic acid, chlorogenic acid, *p*-coumaric acid, ferulic acid, and rosmarinic acid), 4 hydroxybenzoic acids (gallic acid, salicylic acid, syringic acid, and vanillic acid), as well as 2 related compounds (tannic acid and quinic acid) against a panel of 10 bacterial strains. The tested phenolic acids showed a bactericidal action with minimum inhibitory concentration (MIC) values in the range of 0.625 to 10.0 mg/mL. A negative correlation between an increase in the polarity of the molecule and antibacterial effect was observed. At minimum bactericidal concentration (MBC), phenolic acids prevent an increase in extracellular pH by halting bacterial metabolism. Under bacterial metabolism-induced alkaline conditions, caffeic, chlorogenic, rosmarinic, and gallic acids change color to brown or green at sub-MIC concentrations. Caffeic and chlorogenic acids induce filamentation of planktonic *P. aeruginosa*. Tested phenolic compounds reduce biofilm biomass in the range of 10% to 90%, with Gram-positive biofilms being more susceptible. SEM imaging revealed a significantly diminished biofilm EPS with damaged cell structure after phenolic acid treatment. Out of the tested compounds, *p*-coumaric acid and salicylic acid show the most promise as antibacterial agents against bacterial species characteristic of wound infections.

**IMPORTANCE:**

The search for new antibacterial agents is one of the highest healthcare priorities. Phenolic acids are increasingly researched alone and as part of various drug delivery systems for wound care applications. However, to date, the available literature on the antibacterial effects of these compounds is rather fragmented and outdated, with unreproducible results. In this study, we link the physicochemical properties and structure of the molecules with their antibacterial and antibiofilm potential, investigate phenolic acid’s impact on the bacterial metabolism-induced extracellular pH change, and use SEM imaging to characterize the morphological changes of both planktonic bacteria and biofilms in response to phenolic acid treatment. Additionally, we provide an assessment of the compound ADMET properties with respect to their potential topical application. The data can be used in the future to test potential synergic effects with antibiotics, develop drug delivery systems, and investigate the efficacy of these agents against multi-resistant bacterial strains.

## INTRODUCTION

Natural phenolic acids are one of the most widely distributed groups of plant polyphenols, concentrating mainly in the skins of fruits, various grains, and vegetable leaves (1). Although they are typically found in bound forms, both soluble (e.g., conjugates with sugars) and insoluble (e.g., bound to the cell wall), free compounds can occur as well (1, 2). Phenolic acids can be divided into two main classes—hydroxycinnamic acids and hydroxybenzoic acids. The structure of these compounds is based on a phenol ring. The main difference between groups is the location of the carboxyl group (-COOH). For hydroxybenzoic acids, the carboxyl group is directly connected to the phenol ring, while in hydroxycinnamic acids, it is separated by a carbon-carbon double bond (3). The molecules within groups differ mainly by hydroxyl (-OH) or alkoxyl (-O-R) substituents (3).

Phenolic acids are mainly known for their potent antioxidant activity, comparable to that of vitamins (4, 5). Research indicates that the antioxidative properties of these compounds could play a key role in oxidative-stress-related diseases such as cancer, cardiovascular, and metabolic disorders (1, 3). However, the antimicrobial activity of phenolic acids has been generally under-researched. To date, there are only a few studies where the antibacterial activity of several phenolic acids against multiple pathogens is compared in a single study, e.g., Cueva et al. (6), Sánchez-Maldonado et al. (7), Borges et al. (8), and Sanhueza et al. (9). Most studies regarding phenolic acids investigate either a single compound or a single pathogen by applying various methodologies, therefore making data comparison difficult. Furthermore, available data on the antibiofilm activity of phenolic acids are even more scarce and fragmented.

Due to the known antioxidant activity and possible antibacterial potential, they could be useful agents in wound care, including wound infection treatment. Both acute and chronic wound infections have been associated with high morbidity and mortality (10). The treatment options, especially in the case of chronic wounds, are severely hindered by the development of antimicrobial resistance, comorbidities like diabetes mellitus, and the presence of biofilms (10, 11). Compared to planktonic bacteria, biofilms can be 500 to even 1,000 times more tolerant to antimicrobials (11, 12). Moreover, microorganisms in biofilms communicate using a quorum-sensing system, consequently, optimizing metabolic processes, proliferation, and virulence (13). Due to the physical barrier provided by the extracellular polymeric substances (EPS), the penetration of drugs and immune cells is significantly decreased. Biofilms are present in more than 60% of chronic wound infections, indicating the severity of the situation (14).

According to a study by Puca et al., the most common Gram-negative species identified in wound infections are *Pseudomonas aeruginosa* (*P. aeruginosa*), *Escherichia coli* (*E. coli*), and *Proteus mirabilis* (*P. mirabilis*), while the most prevalent Gram-positive bacterial species is *Staphylococcus aureus* (*S. aureus*) (15). In case of burn infections, one of the main pathogens found is *Klebsiella pneumoniae* (*K. pneumoniae*) (16). The cell wall differs between Gram-positive and Gram-negative bacteria. Gram-positive bacteria have a thick cell wall consisting of a peptidoglycan layer measuring from 30 to 100 nm or more, while the peptidoglycan layer of Gram-negative bacteria is only a few nanometers wide (17). The Gram-positive cell wall also contains lipoteichoic and teichoic acids. Opposed to Gram-positive bacteria, Gram-negative ones have an outer membrane, to which lipopolysaccharides and porin channels are connected (17, 18). These differences contribute to bacterial susceptibility to antibacterial agents and the mechanism by which bacterial inhibition is achieved.

**Fig 1:**
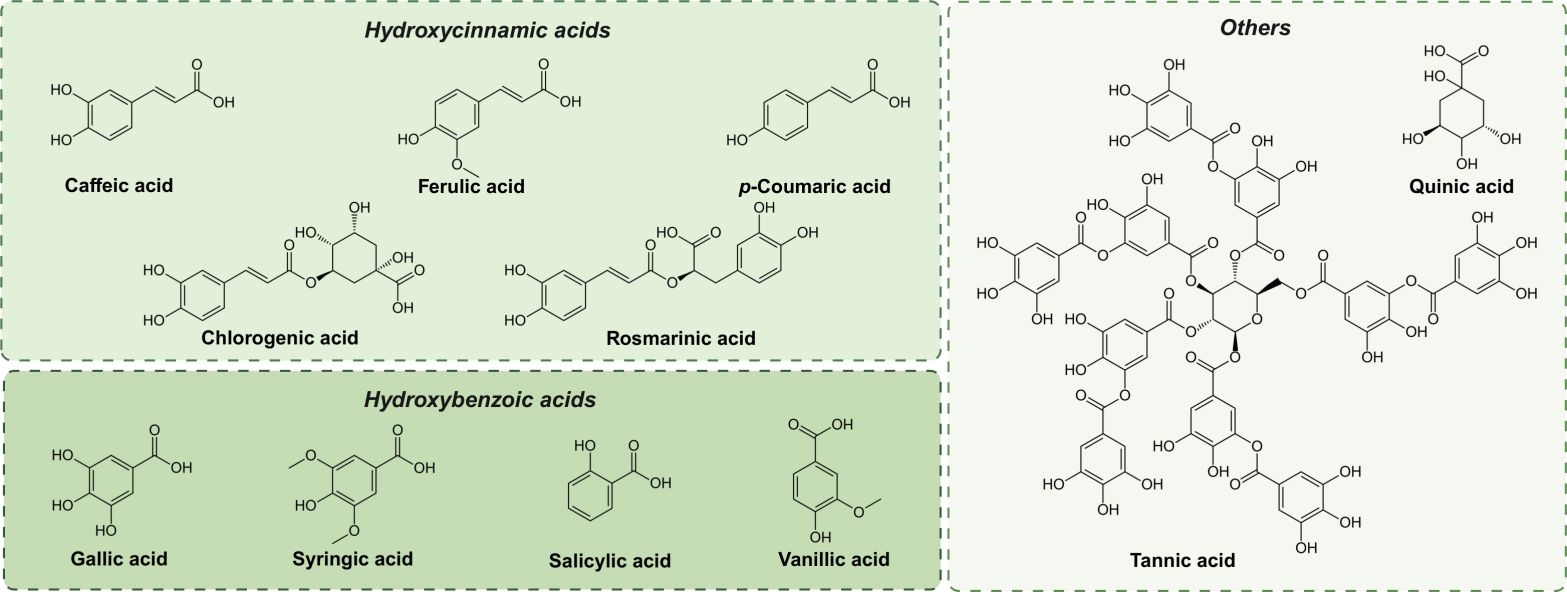
The classification and chemical structures of compounds used in this study. Created in BioRender. Bārzdiņa, A. (2026) https://BioRender.com/ovupq0x.

The aim of this study was to investigate the antibacterial and antibiofilm effects of 5 hydroxycinnamic acids (caffeic acid, chlorogenic acid, *p*-coumaric acid, ferulic acid, and rosmarinic acid), 4 hydroxybenzoic acids (gallic acid, salicylic acid, syringic acid, and vanillic acid), as well as tannic acid and quinic acid against a panel of 10 bacterial strains (*S. aureus* ATCC 6538, *S. aureus* ATCC 25923, *S. epidermidis* ATCC 12228, *B. subtilis* ATCC 6633, *E. coli* ATCC 35218, *E. coli* ATCC 25922, *P. aeruginosa* ATCC 27853, *K. quasipneumoniae* ATCC 700603, *K. pneumoniae* ATCC 10031, *P. mirabilis* ATCC 43071). The compounds were chosen due to their abundant presence in nature and various chemical structures, representing different classes of phenolic acids. The chemical structures of the compounds analyzed in this study can be found in Fig. 1. Changes in bacterial and biofilm morphology due to exposure to these compounds were documented using scanning electron microscopy (SEM). The extracellular pH and color changes before and after microbial growth were explored. *In silico* prediction tools were used to obtain the physicochemical, pharmacokinetic, and toxicity profiles of the tested acids to evaluate their applicability for wound infection treatment. To the best of our knowledge, this is the most comprehensive evaluation of phenolic acid antibacterial and antibiofilm properties against bacterial species characteristic of wound infections, providing an interdisciplinary approach that links the physicochemical and pharmacokinetic properties of the compounds to their antibacterial potential.

## MATERIALS AND METHODS

### Materials and reagents

Caffeic acid (IUPAC: (*E*)-3-(3,4-dihydroxyphenyl)prop-2-enoic acid; ≥98% (HPLC)), tannic acid (IUPAC: [2,3-dihydroxy-5-[[(2*R*,3*R*,4*S*,5*R*,6*S*)-3,4,5,6-tetrakis[[3,4-dihydroxy-5-(3,4,5-trihydroxybenzoyl)oxybenzoyl]oxy]oxan-2-yl]methoxycarbonyl]phenyl] 3,4,5-trihydroxybenzoate; ACS reagent), *p*-coumaric acid (IUPAC: (*E*)-3-(4-hydroxyphenyl)prop-2-enoic acid; ≥98% (HPLC)), vanillic acid (IUPAC: 4-hydroxy-3-methoxybenzoic acid; ≥97% (HPLC)), syringic acid (IUPAC: 4-hydroxy-3,5-dimethoxybenzoic acid; ≥98% (HPLC)), D(-)-quinic acid (IUPAC: trans-(3*R*,5*R*)-1,3,4,5-tetrahydroxycyclohexane-1-carboxylic acid; 98%), ferulic acid (IUPAC: (*E*)-3-(4-hydroxy-3-methoxyphenyl)prop-2-enoic acid; pharmaceutical secondary standard) were purchased from Sigma-Aldrich (St. Louis, MO, USA). Chlorogenic acid (IUPAC: (1*S*,3*R*,4*R*,5*R*)−3-[(*E*)-3-(3,4-dihydroxyphenyl)prop-2-enoyl]oxy-1,4,5-trihydroxycyclohexane-1-carboxylic acid; 98%) was purchased from BLDpharm (Reinbek, Germany). Gallic acid (IUPAC: 3,4,5-trihydroxybenzoic acid; primary reference standard) was purchased from PhytoLab (Vestenbergsgreuth, Germany). Rosmarinic acid (IUPAC: (2*R*)-3-(3,4-dihydroxyphenyl)-2-[(*E*)-3-(3,4-dihydroxyphenyl)prop-2-enoyl]oxypropanoic acid; ≥98%) was purchased from Ambeed (Arlington, IL, USA). Salicylic acid (IUPAC: 2-hydroxybenzoic acid; ≥99%, Ph. Eur) was bought from Carl Roth GmbH & Co (Karlsruhe, Germany). Dimethyl sulfoxide (DMSO) (≥99.9%, sterile filtered) was purchased from Sigma Aldrich (St. Louis, MO, USA). All other reagents were of analytical grade. Due to the limited solubility of phenolic acids, to prepare stock solutions, the compounds were first dissolved in pure DMSO and then diluted with distilled water. The stock solutions of chlorogenic acid, rosmarinic acid, gallic acid, quinic acid, and tannic acid contained 10% DMSO. The stock solutions of caffeic acid, *p*-coumaric acid, ferulic acid, salicylic acid, syringic acid, and vanillic acid contained 40% DMSO. A detailed description of the stock concentrations and their preparation methods can be found in Table S1. Before microbiological testing, the stock solutions were diluted with broth. Solvent controls matching the DMSO content of the diluted stock solutions were used across all testing methods.

*S. aureus* ATCC 25923, *S. aureus* ATCC 6538, *S. epidermidis* ATCC 12228, *B. subtilis* ATCC 6633, *E. coli* ATCC 25922, *E. coli* ATCC 35218, *P. aeruginosa* ATCC 27853, *P. mirabilis* ATCC 43071, and *K. quasipneumoniae* ATCC 700603 were obtained from Liofilchem (Roseto degli Abruzzi, Italy). *K. pneumoniae* ATCC 10031 was purchased from Microbiologics (St. Cloud, MN, USA).

### Determination of minimum inhibitory concentration and minimum bactericidal concentration

To determine the minimum inhibitory concentration (MIC) values, the broth microdilution method was performed according to ISO 20776-1:2019 (19) and EUCAST guidelines (20).

Briefly, in a 96-well plate (Sarstedt, Germany), twofold serial dilutions of tested compounds using Mueller-Hinton broth (MHB) (Oxoid, UK) were carried out in a 50 µL volume. Next, 50 µL of bacterial suspensions was added to the wells to reach the concentration of 5 × 10^5^ CFU/mL followed by careful mixing. The plate was wrapped with PARAFILM M to avoid evaporation and cross-contamination and incubated under static conditions in a thermostat (Memmert GmbH + Co KG, Schwabach, Germany) at 37°C for 24 h. Negative (sterile broth), positive (broth inoculated with bacteria), and solvent (DMSO) controls were used. After the incubation, the plates were visually examined for bacterial growth. The lowest concentration that completely inhibited visible bacterial growth was determined to be the MIC.

To determine the minimum bactericidal concentration (MBC) values of compounds, 10 µL was taken from wells one dilution below the MIC and all above the MIC and cultivated for 24 h at 37°C on Mueller-Hinton agar (Biomaxima, Poland). The lowest concentration at which all microbial growth was completely inhibited was determined to be the MBC.

### Determination of extracellular pH and color change

The pH of wells at the concentrations 2 × MIC, MIC, and 1/2 × MIC was measured at the end of the MIC assay using a pH meter (Mettler Toledo, Columbus, OH, USA) equipped with a HI1330P pH Electrode (Hanna Instruments, Inc., Woonsocket, RI, USA). To determine the pH at the start of the MIC assay, sterile broth instead of bacterial suspension was added to the twofold diluted compounds. It was preliminarily determined that before incubation, the inoculation of bacteria did not affect the pH compared to sterile broth. The pH changes during the assay (∆pH) were calculated by subtracting the pH at the starting point from the pH measured at the end of the MIC assay at the aforementioned concentration levels.

Additionally, the plates were photographed at the end of the MIC assay, and the HEX color code of wells was then obtained using an online tool (21). Wells, where no visible color change compared to sterile broth was observed, were marked with the same HEX color code as broth. To visualize the color differences, the HEX color codes were then used to create a figure in BioRender.com.

### Determination of biofilm-forming capacity

After susceptibility testing, the biofilm-forming capacity of strains was evaluated according to a study by Stepanović et al. (22) using the crystal violet staining assay.

Overnight-grown bacterial cultures were inoculated in a broth (tryptic soy broth [TSB] [Oxoid, UK ]+ 1% glucose for Gram-positive; Luria-Bertani [LB] [Liofilchem, Italy] for Gram-negative bacteria) and incubated for 24 h at 37°C, followed by a 1:100 dilution. This inoculation procedure was used for all biofilm-related assays. In flat-bottom 96-well plates (Sarstedt, Germany), 100 µL of bacterial suspension was added to 100 µL of broth. The plates were wrapped in PARAFILM M and incubated under static conditions in a thermostat at 37°C for 24 h. Sterile broth was used as a negative control.

After the incubation period, all contents of the plates were removed, and the wells were washed with 250 µL of 0.9% saline solution three times. After air-drying, 200 µL of aqueous 0.1% crystal violet solution (Deltalab, Spain) was added and left to stain for 15 min. Then, the crystal violet was discarded, and all the wells were washed with 250 µL of distilled water three times. Lastly, 200 µL of 96% ethanol was added to dissolve the bound crystal violet. The optical density (OD) was measured at 570 nm using a microplate reader (Tecan Infinite F50, Männedorf, Switzerland).

The average OD of the negative control and strains, as well as the cut-off value (ODc), was calculated. The bacterial strains were categorized into the following groups: weak biofilm producer (ODc < OD ≤ 2 × ODc), moderate biofilm producer (2 × ODc < OD ≤ 4 × ODc), and strong biofilm producer (4 × ODc < OD) (22).

### Determination of biofilm prevention concentration and biofilm tolerance factor-prevention

To test whether phenolic acids can prevent the development of bacterial biofilms, twofold dilutions in a 100 µL volume using either TSB + 1% glucose or LB were performed. Furthermore, 100 µL of bacterial suspension (see previous section for inoculation) was added, and the plates were incubated at 37°C for 24 h. The wells were washed, stained, and measured following the steps provided in the previous section (determination of biofilm-forming capacity). The biofilm prevention concentration (BPC) was defined as the lowest concentration that prevented ≥90% of biofilm growth compared to the positive control (23). The BPC values were further used to calculate the ratio between the BPC and MIC, also known as biofilm tolerance factor-prevention (BTF-P) (24).

### Biofilm eradication assay

To test the ability of phenolic acids to reduce the biomass of already preformed biofilms, 100 µL of bacterial suspension was seeded in 100 µL of broth and incubated in static conditions at 37°C for 24 h. After incubation, the content of the wells was carefully aspirated and discarded. The wells were washed thrice with 200 µL of 0.9% NaCl solution. Next, fresh broth (for positive and negative control) or phenolic acid solutions in a concentration range of 2 × MIC to 1/2 × MIC were added, and the plates were incubated at 37°C for 24 h. Subsequently, the plates were washed, stained, and measured as described before. The biofilm biomass reduction percentage was calculated using the following equation:


$$\text{Biomass reduction }\left(\text{\%}\right)\text{= }\frac{{\text{(OD}}_{\text{ positive control}}\text{-}{\text{ OD}}_{\text{negative control}}\text{) - }{\text{(OD}}_{\text{sample}}\text{- }{\text{OD}}_{\text{negative control}}\text{)}}{\text{(}{\text{OD}}_{\text{positive control}}\text{-}{\text{ OD}}_{\text{negative control}}\text{)}}\text{×100}$$

where OD is the optical density, the positive control is untreated bacterial biofilm, the negative control is sterile broth, and the sample is treated biofilm with phenolic acids.

### Scanning electron microscopy

The Calgary Biofilm Device (CBD) (25) was used to prepare the biofilms for SEM imaging. The biofilm assays were carried out as described previously. After the incubation, the top of the plate containing the pegs was carefully removed and washed thrice with a phosphate-buffered solution (PBS) (pH 7.4). Next, the biofilms were fixed by dipping the pegs into a 2.5% glutaraldehyde solution in PBS for 4 h. Afterward, dehydration of the biofilm was performed by dipping the pegs into a series of increasingly concentrated ethanol (30%, 50%, 70%, 90%, and 96%) with each stage lasting 15 min. The last stage was repeated twice. Finally, the top part of the plate was placed in a desiccator and stored until imaging. Before imaging, the polystyrene pegs were carefully cut off and attached to conductive carbon adhesive tape-covered SEM specimen stubs. The samples were coated with a 10 nm layer of carbon using a LEICA EM ACE 200 coater. The morphology of samples was examined using a high-resolution field emission scanning electron microscope SEM/STEM (Verios 5 UC, Thermo Fisher). Secondary electron SEM images were acquired utilizing through-lens (TLD), operated at 5 kV. To prevent charging and sample damage, a low current (50 pA) and vector scanning approach with a dwell time of 50 ns was employed. The obtained images were processed using the ImageJ software (26).

### ADMET (absorption, distribution, metabolism, excretion, toxicity) analysis

The physicochemical and pharmacokinetic parameters of phenolic acids were predicted using the SwissADME (27) online tool. The SMILES identifier and pKa values of compounds were obtained from the PubChem (28) database. Toxicity parameters (AMES toxicity, hERG I and II inhibition, hepatotoxicity, skin sensitization) were obtained from the pkCSM (29) online tool. Additional toxicity information (LD50 values and cytotoxicity) was obtained from the ProTox 3.0 webserver (30).

### Statistical analysis and visualizations

All experiments were carried out in triplicate. Data analysis has been carried out on Microsoft Excel (Microsoft 365) and BioRender Graph software. Data are shown as mean ± SD. Figures were created using BioRender.com. Chemical structures of the tested molecules were obtained using the ChemRender Beta function in BioRender.com. SMILES string was used to search for molecules in the PubChem repository.

## RESULTS AND DISCUSSION

### Sensitivity testing and structure-function relationships of tested compounds

In the hydroxycinnamic acid class, the MIC values ranged from 0.625 mg/mL to 10 mg/mL, while for hydroxybenzoic acids, they ranged from 1.25 mg/mL to 10 mg/mL, respectively (Table 1).

**TABLE 1:** The minimum inhibitory concentrations (MIC) of tested compounds (mg/mL)

Compound	MIC (mg/mL)									
	Gram-positive strains				Gram-negative strains					
	*S. aureus* ATCC 6538	*S. aureus* ATCC 25923	*S. epidermidis* ATCC 12228	*B. subtilis* ATCC 6633	*E. coli* ATCC 35218	*E. coli* ATCC 25922	*P. aeruginosa* ATCC 27853	*K. quasipneumoniae* ATCC 700603	*K. pneumoniae* ATCC 10031	*P. mirabilis* ATCC 43071
Caffeic acid	2.5	2.5	2.5	2.5	5.0	2.5	2.5	2.5	2.5	2.5
Chlorogenic acid	10.0	10.0	5.0	5.0	10.0	10.0	10.0	10.0	10.0	10.0
*p*-Coumaric acid	1.25	1.25	1.25	0.625	2.5	2.5	2.5	1.25	1.25	2.5
Ferulic acid	1.25	2.5	2.5	1.25	2.5	2.5	5.0	2.5	2.5	2.5
Rosmarinic acid	5.0	5.0	5.0	5.0	10.0	5.0	5.0	5.0	10.0	5.0
Gallic acid	5.0	10.0	5.0	2.5	5.0	5.0	2.5	5.0	5.0	5.0
Salicylic acid	1.25	1.25	1.25	1.25	1.25	1.25	1.25	1.25	1.25	1.25
Syringic acid	2.5	2.5	2.5	2.5	5.0	2.5	2.5	5.0	5.0	2.5
Vanillic acid	1.25	2.5	2.5	2.5	2.5	2.5	2.5	2.5	2.5	2.5
Quinic acid	5.0	5.0	5.0	2.5	5.0	5.0	2.5	5.0	5.0	5.0
Tannic acid	0.313	0.078	0.039	>10.0	>10.0	>10.0	>10.0	>10.0	>10.0	>10.0

In the hydroxycinnamic acid class, the most potent antibacterial activity was shown by *p*-coumaric acid, followed by ferulic acid and caffeic acid. It’s worth mentioning that all of these molecules have at least one free hydroxyl group (-OH) (Fig. 1). The antibacterial potency structure-wise ranges in the following order: 4-hydroxy > 4-hydroxy-3-methoxy > 3,4-dihydroxy. The results suggest that fewer substituents (either hydroxyl or methoxy groups) result in a stronger antibacterial effect. Interestingly, the poorest activity was shown by chlorogenic acid and rosmarinic acid, which are both esters. This indicates an important role of the free carboxyl group in the antibacterial mechanism of hydroxycinnamic acids. The activity of hydroxycinnamic acids is rather strain-specific, and no significant distinction between Gram-positive and Gram-negative strains can be made.

Out of the hydroxybenzoic acid class, salicylic acid demonstrated the strongest antibacterial activity with an MIC of 1.25 mg/mL against all strains, followed by vanillic acid and syringic acid, with gallic acid showing the poorest activity. This indicates a clear correlation between the number of substituents in the hydroxybenzoic acid molecule and the antibacterial effect. The potency follows the order: 2-hydroxy > 4-hydroxy-3-methoxy > 4-hydroxy-3,5-dimethoxy > 3,4,5-trihydroxy. A similar structure-function correlation with more hydroxyl groups resulting in poorer activity of hydroxybenzoic acids was observed by Sánchez-Maldonado when testing the gallic, syringic, protocatechuic, and *p*-hydroxybenzoic acids (7). Overall, the activity of hydroxybenzoic acids against Gram-positive and Gram-negative strains is comparable.

A relationship between polarity and antibacterial activity was observed for both hydroxycinnamic and hydroxybenzoic acids. The more polar the molecule (more hydroxyl and methoxy substituents), the less potent is the inhibitory effect against bacteria. The mechanism could be related to more lipophilic compounds being able to adhere and penetrate the lipophilic bacterial cell membrane. This indicates that at least partly the inhibitory effects are connected to the disruption of cell membrane integrity. This coincides with a study on flavonoids by Yuan et al. (31). A more detailed comparison of the polarity coupled with other physicochemical properties of the tested phenolic acids will follow in section entitled Physicochemical and ADMET properties.

Tannic acid, whose molecule consists of multiple galloyl units, showed a specific, several-fold higher activity against *Staphylococcus* spp. than the other tested compounds. It was inactive against other strains of bacteria. This coincides with a study by Wu et al., where a multiple-fold lower MIC of tannic acid against *Staphylococcus* spp. compared to Gram-negative bacteria was reported (32). A study by Dong et al. suggests that the strong antibacterial effects of tannic acid against *Staphylococcus* spp. may be due to direct binding to peptidoglycan, therefore, disrupting the integrity of the cell wall (33). *B. subtilis*, which is also a Gram-positive strain, can produce tannase, an enzyme that hydrolyzes tannic acid to gallic acid and glucose (34). It may be the reason why tannic acid showed no activity against *B. subtilis*.

Quinic acid, which was chosen due to its presence in the chlorogenic acid molecule, showed on average twofold lower MIC concentrations than chlorogenic acid. The MIC values (2.5 to 5 mg/mL) are consistent with those obtained by Bai et al. (35).

Overall, equal MIC values with few exceptions were obtained between different strains of the same bacterial species. Caffeic acid, rosmarinic acid, and syringic acid had twofold higher MIC values against *E. coli* ATCC 35218 than *E. coli* ATCC 25922. Ferulic acid, gallic acid, and vanillic acid had twofold higher MIC values against *S. aureus* ATCC 25923 than *S. aureus* ATCC 6538. The observed twofold differences may be due to assay variability. However, it’s necessary to test natural compounds not just on common strains but also on strains with known resistance mechanisms in place. For example, *E. coli* ATCC 35218 is a β-lactamase (TEM-1) producing strain (36). Although the tested compounds do not have a beta-lactam ring, beta-lactamase-producing strains could develop additional resistance mechanisms like the overexpression of efflux pumps or the reduction of porin channels (37).

Although previous studies have reported MIC values of caffeic acid (MIC 256–1,024 µg/mL [38]; MIC 0.625–5 mg/mL [39]), chlorogenic acid (MIC 2–16 mg/mL [40]; MIC 2,047.78–2,048.22 µg/mL [41]; MIC 2.50 mg/mL [42]; MIC 2.56 mg/mL [43]; MIC 2.5–10 mg/mL [44]), ferulic acid (MIC 100–1,250 µg/mL [8]), rosmarinic acid (MIC 625–1,250 µg/mL [45]; MIC 0.3–2.5 mg/mL [46]), gallic acid (MIC 500–2,000 µg/mL [8]; MIC 5 mg/mL [47]; MIC 2 mg/mL [48]), salicylic acid (MIC >500 µg/mL [49]), vanillic acid (MIC 11.80 mM [50]), quinic acid (MIC 2.5–5 mg/mL [35]), tannic acid (MIC 0.12–>7.2 mg/mL) [32]), a direct comparison of the MIC values cannot be made since methodology in terms of specific strain, solvent types and concentration, inoculum, etc., is not consistent. However, in most studies, the MIC values are in the 1–10 mg/mL range, therefore coinciding with our results.

To test at which concentrations the tested compounds not only inhibit visible bacterial growth but also kill 99.9% of inoculated bacteria, the MBC assay was performed. The MBC values can be found in Table 2, while the ratio between MBC/MIC can be found in Table S2. The lowest MBC values can be seen for salicylic acid and *p*-coumaric acid, which are in the 1.25 to 2.5 mg/mL range. For all phenolic acids, MBC is equal to or twofold higher than MIC. Only *p*-coumaric acid against *B. subtilis* has an MBC fourfold higher than MIC. In cases where the MBC is higher than MIC, the bacteria may have put in place various mechanisms to protect themselves, e.g., the production of phenolic acid decarboxylase (PAD). *B. subtilis* can produce a PAD that decarboxylates ferulic, *p*-coumaric, and caffeic acids into 4-vinyl derivatives (51). The vinyl derivatives are less toxic to Gram-positive bacteria compared to the phenolic acids (52). In this study, the MBCs of caffeic, *p*-coumaric, and ferulic acid are two- to fourfold higher than the MIC.

**TABLE 2:** The minimum bactericidal concentrations (MBC) of tested compounds (mg/mL)

Compound	MBC (mg/mL)									
	Gram-positive strains				Gram-negative strains					
	*S. aureus* ATCC 6538	*S. aureus* ATCC 25923	*S. epidermidis* ATCC 12228	*B. subtilis* ATCC 6633	*E. coli* ATCC 35218	*E. coli* ATCC 25922	*P. aeruginosa* ATCC 27853	*K. quasipneumoniae* ATCC 700603	*K. pneumoniae* ATCC 10031	*P. mirabilis* ATCC 43071
Caffeic acid	2.5	5.0	2.5	5.0	5.0	2.5	2.5	5.0	5.0	5.0
Chlorogenic acid	10.0	10.0	10.0	5.0	10.0	10.0	10.0	10.0	10.0	10.0
*p*-Coumaric acid	2.5	2.5	2.5	2.5	2.5	2.5	2.5	2.5	2.5	2.5
Ferulic acid	2.5	2.5	2.5	2.5	5.0	2.5	5.0	5.0	5.0	5.0
Rosmarinic acid	10.0	10.0	10.0	5.0	10.0	5.0	10.0	10.0	10.0	5.0
Gallic acid	5.0	10.0	5.0	5.0	10.0	5.0	5.0	5.0	5.0	10.0
Salicylic acid	1.25	2.5	1.25	1.25	2.5	2.5	2.5	2.5	2.5	2.5
Syringic acid	5.0	5.0	5.0	2.5	5.0	5.0	5.0	5.0	5.0	5.0
Vanillic acid	2.5	2.5	2.5	2.5	5.0	5.0	5.0	2.5	2.5	5.0
Quinic acid	5.0	5.0	5.0	2.5	5.0	5.0	5.0	5.0	5.0	5.0
Tannic acid	10.0	0.625	1.25	>10.0	>10.0	>10.0	>10.0	>10.0	>10.0	>10.0

As the ratio of MBC/MIC ≤ 4, the antibacterial mechanism of action of these phenolic acids is bactericidal (53). The MBC/MIC ratio for tannic acid against the *Staphylococcus* spp. exceeds 4, therefore indicating a bacteriostatic mechanism.

Overall, the antibacterial effect of phenolic acids can be achieved through multiple mechanisms. First, phenolic acids can disrupt the bacterial membrane integrity by hyperacidification. As a result, they can cause a change in bacterial membrane potential, enhance membrane permeability, and induce the leakage of intracellular components (8, 54). Phenolic acids can cause both extracellular and intracellular acidification based on the dissociation level of the molecules. Intracellular acidification in the undissociated form can lead to protein denaturation, damaged membranes, and enhanced potassium efflux (8, 55). Second, phenolic acids can impact the energy metabolism of bacteria, interfering with the respiratory activity, tricarboxylic acid cycle, and others (2). Third, phenolic acids can halt bacterial DNA replication and protein synthesis (2). Nevertheless, the exact mechanism or combination of mechanisms may differ between compounds, and wide generalizations should be avoided. The antibacterial mechanisms of the tested compounds in terms of extracellular acidification, damage to membrane integrity, as well as physicochemical properties connected to the dissociation of molecules will be discussed in the following sections.

### Extracellular pH and color change as indicators of bacterial growth

**Fig 2:**
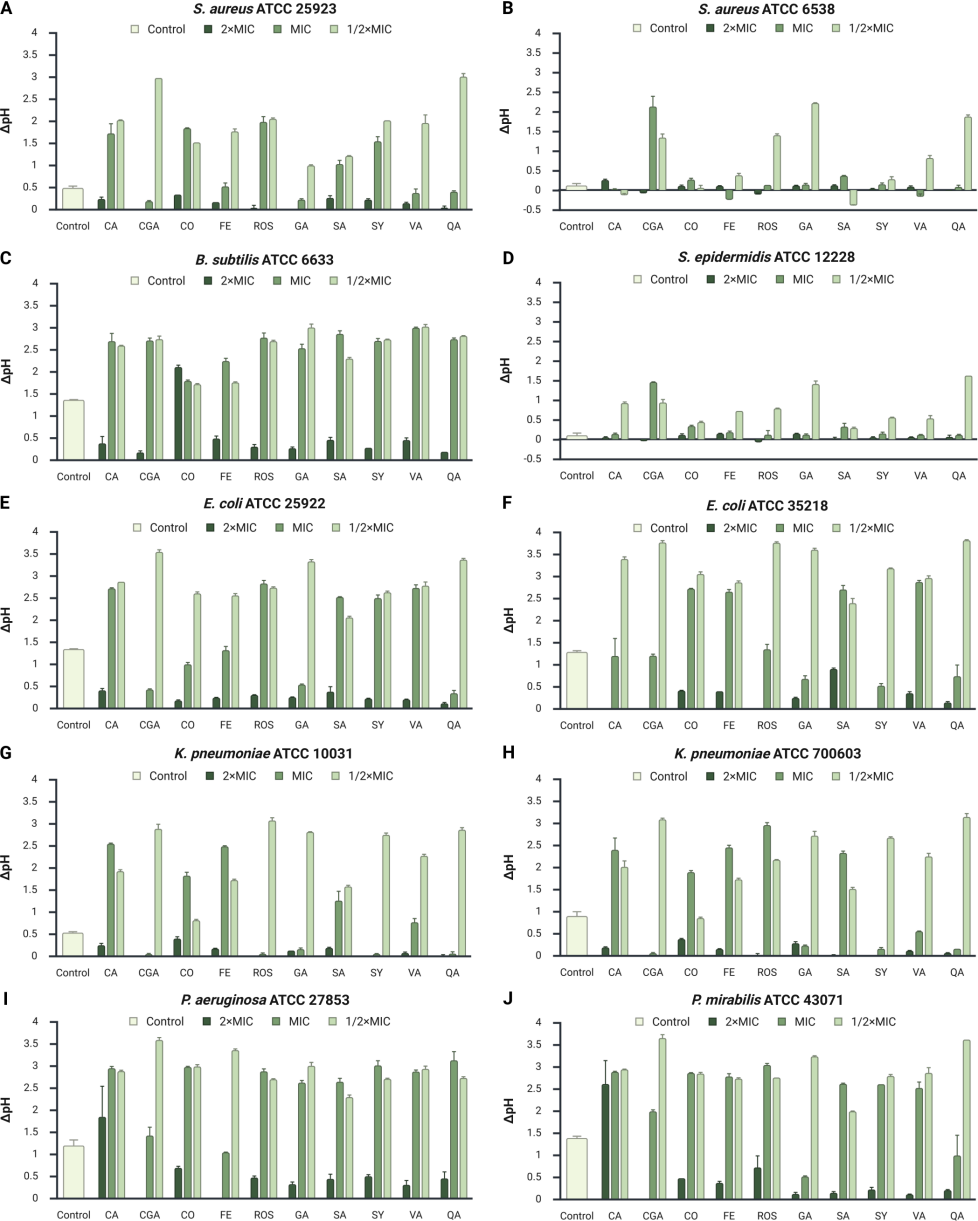
The change in pH (∆pH) during the 24-h MIC assay. (**A**) *S. aureus* ATCC 25923; (**B**) *S. aureus* ATCC 6538; (**C**) *B. subtilis* ATCC 6633; (**D**) *S. epidermidis* ATCC 12228; (**E**) *E. coli* ATCC 25922; (**F**) *E. coli* ATCC 35218; (**G**) *K. pneumoniae* ATCC 10031; (**H**) *K. quasipneumoniae* ATCC 700603; (**I**) *P. aeruginosa* ATCC 27853; (**J**) *P. mirabilis* ATCC 43071. MIC—minimum inhibitory concentration; CA—caffeic acid; CGA—chlorogenic acid; CO*—p*-coumaric acid; FE—ferulic acid; ROS—rosmarinic acid; GA—gallic acid; SA—salicylic acid; SY—syringic acid; VA—vanillic acid; QA—quinic acid. Created in BioRender.com. Bārzdiņa, A. (2026) https://BioRender.com/v38i9ha.

To investigate if there is a relationship between the antibacterial activity and change in the extracellular pH, the pH of the media of wells before and after the MIC assay was measured. Due to the poor antibacterial activity against the majority of tested strains (MIC > 10 mg/mL), tannic acid was excluded from further evaluation. The average pH of controls and compounds before and after 24-h incubation can be found in Table S3. The change in pH (∆pH) during the MIC assay is visualized in Fig. 2.

Before the start of incubation, the extracellular pH in the media of bacterial controls was neutral (7.5 ± 0.1). After incubation and bacterial growth, it was determined that extracellular pH increased for all strains, indicating that through metabolism, the bacteria can modify the pH of the extracellular environment. *Staphylococcus* spp. showed an external pH increase of less than 0.5, while strains of *B. subtilis*, *E. coli, P. aeruginosa*, and *P. mirabilis* modified the pH more substantially with ∆pH > 1.25. The alkalization of the medium may be due to the production of ammonia or other basic compounds by the bacteria. For none of the strains did the extracellular pH exceed 9. The external pH change could be attributed to changes in metabolic processes combined with rapid adaptation rates (56).

It’s important to add that the initial pH, as well as growth media, can significantly alter the extracellular pH (57, 58). Nevertheless, extracellular pH may provide valuable insight into the bacterial growth and metabolism alteration patterns in the presence of phenolic acids. At the start of the MIC assay, the pH in wells with phenolic acids was acidified (pH 3.86–5.84 at MIC concentration). After the 24-h assay, in most cases, a pH increase was observed to various extents.

At MBC concentrations, which are either equal to MIC or 2 × MIC based on our results, the pH stayed roughly the same. We could hypothesize that through acidification combined with other mechanisms, at the MBC level, bacterial death occurred, and as a result, their metabolism was interrupted. A good example may be the results for *p*-coumaric acid against *B. subtilis*. It is the only combination where the MBC/MIC ratio is 4. Therefore, at the 2 × MIC level, the bacterial metabolism continues, indicated by ∆pH > 2. For comparison, ∆pH ≤ 0.5 was observed for other compounds at the same concentration level.

At the MIC level for all bacteria, the ∆pH varies depending on the MBC of the compound and the extracellular pH change power of the strain. Against strains whose controls had a ∆pH ≥ 1, the pH increased at the MIC level regardless of other factors. At the sub-MIC level, a significant pH increase was observed, reaching or nearing that of the control.

Additionally, the pH measurements indicate that extracellular acidification is not the only mechanism involved in the antibacterial effect. At roughly the same starting pH values and the same strain of bacteria, during the assay, the achieved ∆pH varied significantly between compounds. For instance, against *E. coli* ATCC 25922, at the start of the assay, the pH of caffeic acid at 2 × MIC concentration was 4.66 ± 0.03, and the pH of chlorogenic acid at 1/2 × MIC was 4.68 ± 0.01. After the assay, the pH of caffeic acid was 5.06 ± 0.07, and for chlorogenic acid, it was 8.21 ± 0.04. These results indicate that the metabolism alterations shown by ∆pH are not dependent on the starting pH, but rather on the compound-specific mechanisms that likely combine extracellular acidification, molecule physicochemical parameters, and others. The physicochemical properties of tested molecules will be discussed in detail in section Physicochemical and ADMET properties.

**Fig 3:**
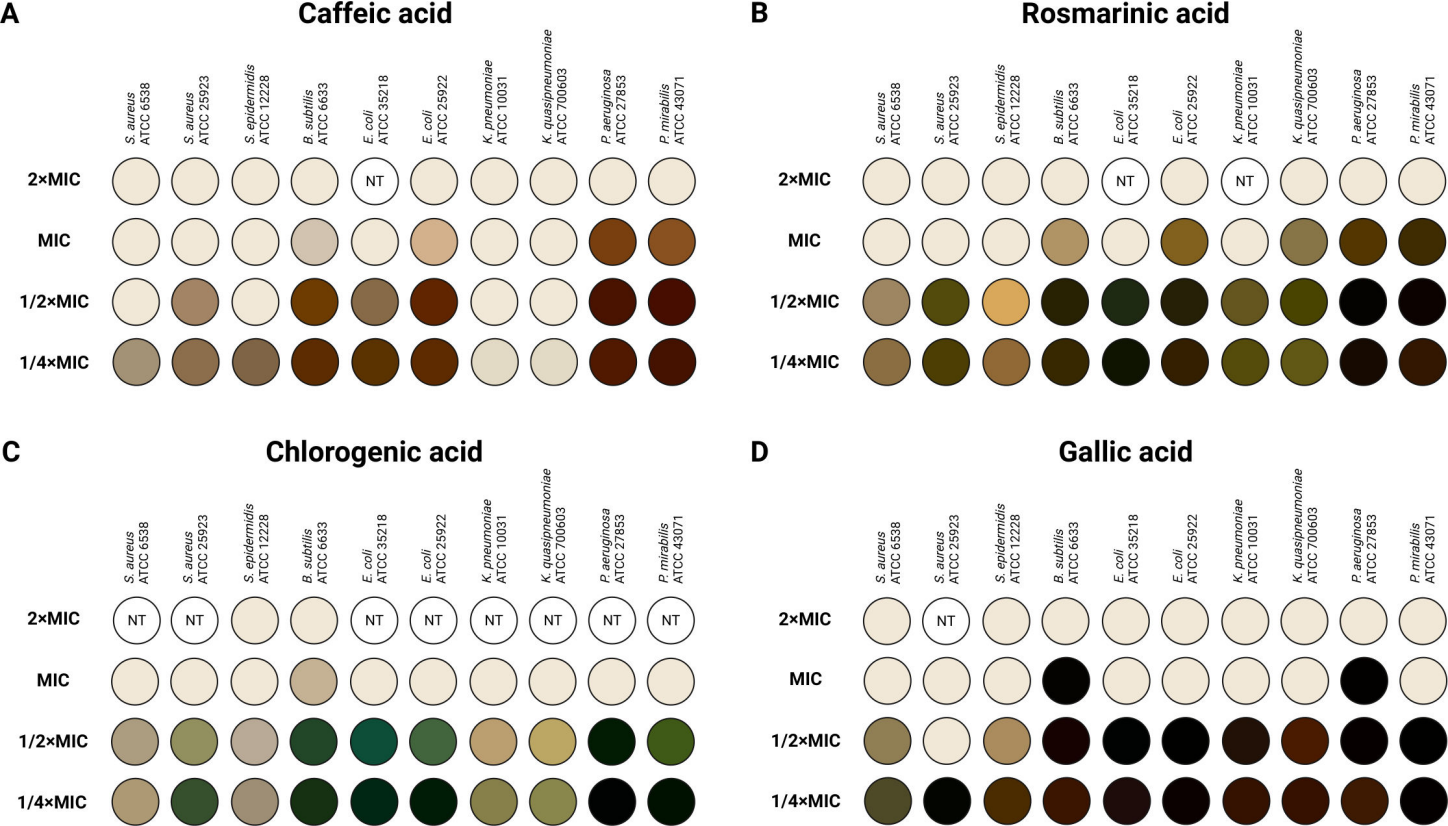
The color of wells after the MIC assay. (**A**) Treated with caffeic acid; (**B**) treated with rosmarinic acid; (**C**) treated with chlorogenic acid; (**D**) treated with gallic acid. MIC—minimum inhibitory concentration; NT—not tested. Created in BioRender. Bārzdiņa, A. (2026) https://BioRender.com/63zn8yk.

Regarding the metabolites responsible for the change in pH, the observed color change of four tested phenolic acids (caffeic acid, chlorogenic acid, rosmarinic acid, and gallic acid) could be an indication of an excess of amino compounds present in the media under alkaline conditions. The observed color changes are visualized in Fig. 3, while the photographs are available in Table S4. The color change for these phenolic acids is dependent on the pH of the media, with coloring occurring only under alkaline conditions. The most intense color change is seen against strains with strong extracellular pH change potential*—B. subtilis*, *E. coli*, *P. aeruginosa*, and *P. mirabilis*.

Based on the available literature, the coloring of these compounds occurs due to phenolic acid oxidation to a quinone form either enzymatically or non-enzymatically. As the quinone form is electrophilic and highly reactive, the reaction continues to form colored products (59). For example, the green color observed for chlorogenic acid could be explained by chlorogenic acid oxidation to form chlorogenic acid quinones, which, in turn, can react with amino acids or proteins, forming green-colored compounds like trihydroxy benzacridine derivatives (60). If amino acids are not present, the chlorogenic acid quinones polymerize, giving a brown color (61). Non-enzymatic oxidation is accelerated under alkaline conditions. A catechol or pyrogallol group in a molecule is required for the oxidation to quinone (59). Therefore, only caffeic acid, chlorogenic acid, rosmarinic acid, and gallic acid showed color change, while the other tested compounds did not. The aforementioned mechanism strengthens the hypothesis that the alkalization of the extracellular media could be due to the generation of amino compounds to a varying extent, depending on the strain, subsequently leading to reactions with quinones predominantly in sub-MIC concentrations.

The oxidation of catechol or pyrogallol group to *o*-quinones could relate to the antibacterial mechanism of these molecules through the protein thiol oxidation route (62). The *o*-quinones can covalently bind to protein cysteine thiols using the Michael addition mechanism, therefore forming cysteine-quinone adducts (63). In bacteria, the thiol-dependent redox systems regulate the reactive oxygen species (ROS) levels (64). Cysteine-quinone adducts can disrupt the redox homeostasis in bacteria. As a result, excessive ROS production within bacterial cells could cause cell damage and even lead to bacterial death (64).

The production of colored products under alkaline conditions could also be important for the potential application of these compounds. In chronic wounds, the natural slightly acidic environment (pH 4–6) is disrupted and can reach the pH range of 7.15–8.9 (65, 66). The alkalization of the wound environment is due to the presence of blood and interstitial fluid as well as the ureolytic activity of bacteria, which results in the production of ammonia from urea (67). In this scenario, phenolic acids, which have the catechol or pyrogallol group in their structure, could serve a double purpose. First, these compounds have acidic properties, and at concentrations equal to or higher than MIC, they could help to fight the infection and lower the pH of the infection site. It’s been proven that wound healing is improved after acidification (68). Second, the color change behavior could act as a smart indicator of the pH and therapeutic progress in specialized drug delivery systems such as hydrogels (69, 70). Future metabolic studies on the exact compounds responsible for the increase in extracellular pH and the coloration products should be conducted.

### Antibiofilm effects of phenolic acids

Since preliminary tests showed that *S. epidermidis*, *B. subtilis*, and *P. mirabilis* strains did not form biofilms under the tested conditions, these strains were eliminated from further biofilm testing.

The anti-biofilm properties of phenolic acids and quinic acid were determined against seven strains, out of which six strains were classified as strong biofilm formers (*S. aureus* ATCC 25923, *E. coli* ATCC 25922, *E. coli* ATCC 35218, *K. pneumoniae* ATCC 10031, *K. quasipneumoniae* ATCC 700603, and *P. aeruginosa* ATCC 27853) and one as a weak biofilm former (*S. aureus* ATCC 6538) in accordance with the biofilm production classification system proposed by Stepanović et al. (22). Although the classification was first developed for *Staphylococcus* spp., it has since been applied to a wider range of species, including *E. coli* (71), *P. aeruginosa* (72), and *K. pneumoniae* (73). Based on the available literature, bacterial species included in this study have all been found in wound-related biofilms (15, 16). Since chlorogenic acid and rosmarinic acid form a small precipitate that binds to crystal violet and gives misleadingly high absorption values, the results of these compounds from crystal violet-based assays are not reported.

### Biofilm prevention

The BPC values of tested compounds (Table S5) were obtained to determine what concentration of compound is needed to prevent planktonic bacteria from starting to form bacterial biofilms. The ratio between the BPC and MIC values (BTF-P) can be found in Table S6. The BTF-P values range from 0.5 to 4, with the majority being 0.5 and 1, meaning that sub-MIC or MIC concentrations are sufficient in preventing the start of biofilm development.

**Fig 4:**
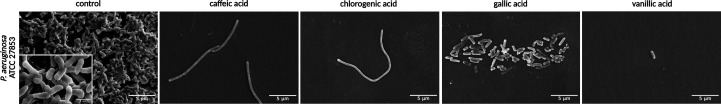
The morphological changes of *P. aeruginosa* ATCC 27853 planktonic bacteria, biofilm prevention assay, scanning electron microscopy (SEM), 5,000× magnification, zoomed in control magnification 25,000×. Scale bar equal to 5 µm.

*P. aeruginosa* ATCC 27853 samples were visualized using SEM (see Fig. 4) to investigate if, at the BPC level, phenolic acids not only prevent biofilm development but also additionally induce morphological changes in the planktonic bacteria. Two hydroxycinnamic acids (caffeic acid and chlorogenic acid) and two hydroxybenzoic acids (vanillic acid and gallic acid) were chosen as representatives. In the *P. aeruginosa* control group, the bacteria have a classic rod shape with a length between 1.2 and 1.8 µm and a width between 0.5 and 0.6 µm. When treated with hydroxybenzoic acids, the bacterial shape does not change, but the cell wall is rough and damaged, especially in the case of gallic acid. In the presence of hydroxycinnamic acids (caffeic acid and chlorogenic acid), bacterial cell filamentation was observed. Filamentation is a known response to environmental or antibiotic-induced stress, where cell division is interrupted while elongation continues (74). The detected filamented bacterial length was in the range of 3.6 to 18.7 µm.

### Biofilm eradication

**Fig 5:**
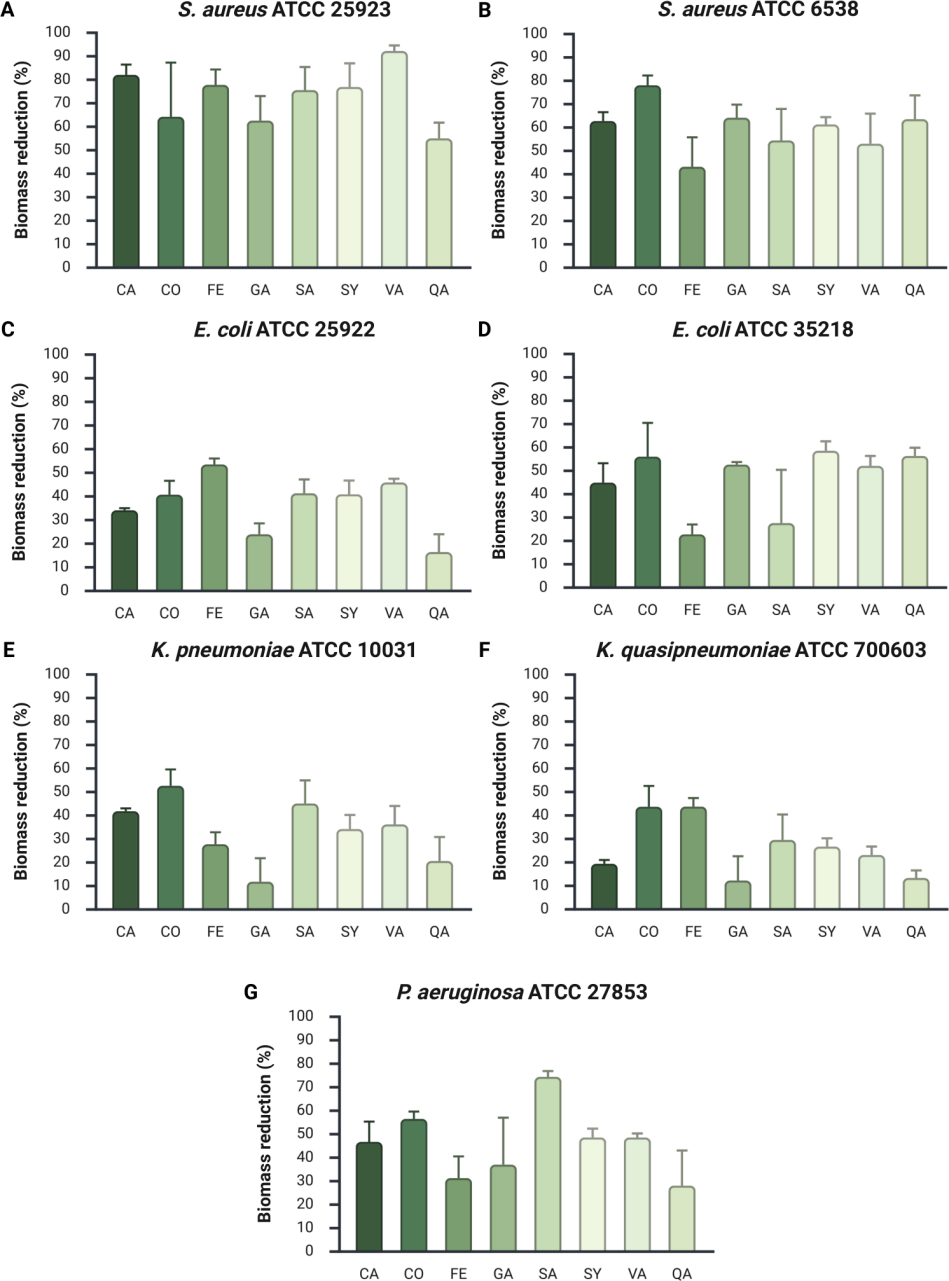
Biofilm biomass reduction (%) at concentration 2 × MIC. (**A**) *S. aureus* ATCC 25923; (**B**) *S. aureus* ATCC 6538; (**C**) *E. coli* ATCC 25922; (**D**) *E. coli* ATCC 35218; (**E**) *K. pneumoniae* ATCC 10031; (**F**) *K. quasipneumoniae* ATCC 700603; (**G**) *P. aeruginosa* ATCC 27853. CA—caffeic acid; CO*—p*-coumaric acid; FE—ferulic acid; GA—gallic acid; SA—salicylic acid; SY—syringic acid; VA—vanillic acid; QA—quinic acid. Created in BioRender. Bārzdiņa, A. (2026) https://BioRender.com/hsropid.

In real-life conditions, biofilm formation has occurred prior to intervention; hence, the reduction in already preformed biofilm biomass in response to treatment at 2 × MIC concentration level was measured (visualized in Fig. 5). At 2 × MIC, the compounds reduced the preformed biofilm biomass in the range of 10% to even 90%.

The tested compounds showed stronger biofilm eradication potential against Gram-positive strains (biomass reduction > 40% for all tested compounds) than Gram-negative ones, possibly due to the structural differences of the bacterial cells. Gram-positive bacteria do not have an outer membrane and are protected by a thick peptidoglycan layer, while Gram-negative bacteria, in addition to a thin peptidoglycan layer, have an outer membrane that contains lipopolysaccharides (75). Against the Gram-negative strains, the biomass reduction was mostly under 50% with a few exceptions. Biofilms of *K. quasipneumoniae* and *K. pneumoniae* were particularly resistant to the treatment. *K. quasipneumoniae* ATCC 700603 is an extended-spectrum β-lactamase (ESBL) (SHV-18) producing strain (76). A study by Dan has revealed that for ESBL-producing *K. pneumoniae* strains, biofilm formation is significantly higher compared to non-ESBL-producing strains (77).

Strain-specific effectiveness of compounds was observed. Against *S. aureus* ATCC 25923, caffeic acid showed the highest biomass reduction (81.8% ± 4.4%) out of the tested hydroxycinnamic acids and vanillic acid (91.9% ± 2.4%) out of hydroxybenzoic acids, respectively. This coincides with a previous study, where caffeic acid reduced 80% of *S. aureus* ATCC 25923 biofilm (78). Quinic acid was less active compared to the phenolic acids. Against *S. aureus* ATCC 6538, all compounds showed comparable biofilm biomass reduction, with *p*-coumaric acid having the highest (77.8% ± 4.2%) and ferulic acid the lowest (42.9% ± 12.6%) reduction of all compounds. Interestingly, against *E. coli* ATCC 25922, ferulic acid reduced 53.2% ± 2.6% of biofilm biomass, while against *E. coli* ATCC 35218, it reduced only 22.5% ± 4.2%, respectively. On the contrary, for other compounds, the activity between these strains was comparable or increased against *E. coli* ATCC 35218. Gallic acid and quinic acid showed the weakest, while *p*-coumaric acid showed the strongest activity against *Klebsiella* spp. Against *P. aeruginosa* ATCC 27853, the strongest biomass reduction capacity was demonstrated by salicylic acid (74.1% ± 2.5%) and *p*-coumaric acid (56.2% ± 3.1%).

**Fig 6:**
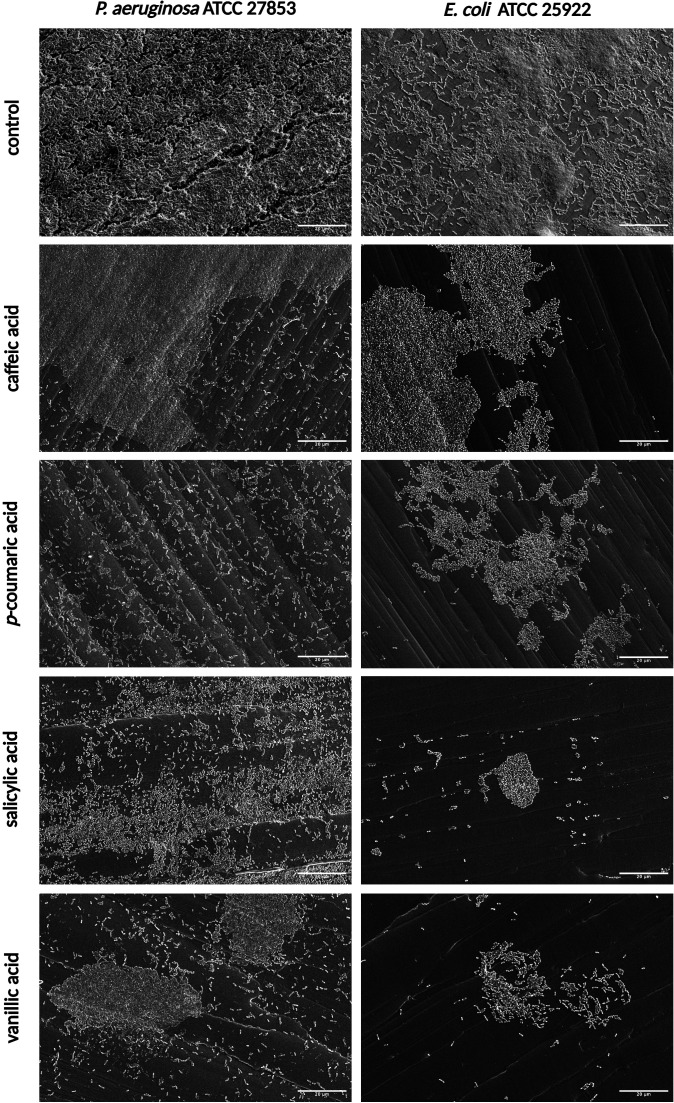
*P. aeruginosa* ATCC 27853 and *E. coli* ATCC 25922 biofilms with and without phenolic acid treatment, biofilm eradication assay, scanning electron microscopy (SEM), 1,000× magnification. Scale bar equal to 20 µm.

**Fig 7:**
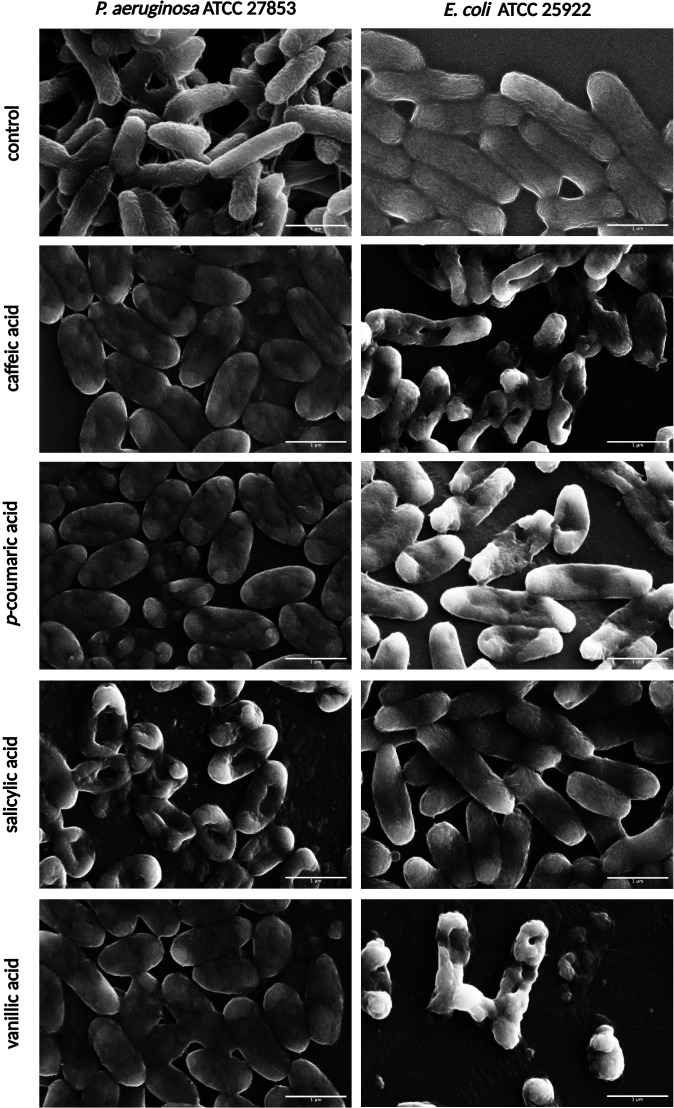
*P. aeruginosa* ATCC 27853 and *E. coli* ATCC 25922 bacterial morphology with and without phenolic acid treatment, biofilm eradication assay, scanning electron microscopy (SEM), 25,000× magnification. Scale bar equal to 1 µm.

The effect of the most promising phenolic acids on *E. coli* and *P. aeruginosa* biofilms was further investigated using SEM (Fig. 6 and 7). *P. aeruginosa* ATCC 27853 biofilm is especially dense, with multiple layers of bacteria forming a 3D structure covering all of the visual field. While the *E. coli* biofilm was not as thick as the one from *P. aeruginosa*, in some areas, bacterial stacking with closely packed bacteria was observed. Compared to control, the thickness and bacterial density of the biofilm had decreased when treated with phenolic acids, leaving either a single layer of biofilm or scattered planktonic bacteria (Fig. 6).

Upon zooming in, the morphology of single bacteria was changed (Fig. 7). For the *P. aeruginosa* control, an excess of EPS can be seen, with which the bacteria are interconnected. On the contrary, for the treated bacteria, a lack of EPS is observed with the bacteria cells being significantly damaged (e.g., salicylic acid), and the shape of the bacteria is rounded and more oval than the rod shape of the *P. aeruginosa* control. For *E. coli*, we can see that the initial shape is retained when treated with phenolic acids. However, in the presence of caffeic acid, *p*-coumaric acid, and vanillic acid, large holes in the cell wall can be observed, indicating leakage of intracellular components and subsequent bacterial death.

When analyzing the biofilm eradication results, we have to consider that the biofilm was grown for 24 h before the introduction of treatment. For more mature biofilms, it is expected that the biomass reduction would be less pronounced. The anti-biofilm effects theoretically could be improved by increasing the concentration of the antibacterial agents, since for antibiotics, the concentration needed to fully eradicate a bacterial biofilm is often even hundreds of times higher than MIC. However, most phenolic acids are not freely soluble in water. As in this study, the stock solutions are first made in various concentrations of DMSO and then further diluted with broth to eliminate the chance of solvent impacting the results. An increase in phenolic acid concentration would come hand in hand with an increase in solvent concentration. Therefore, the phenolic acid solubility issues have to be taken into consideration when thinking about the possible application of these compounds.

### Physicochemical and ADMET properties

*In silico* predictions of physicochemical (Table 3), pharmacokinetic (Table 4), and toxicity (Table 5) properties were obtained to evaluate the applicability of phenolic acids as antibacterial agents.

**TABLE 3:** The predicted physicochemical properties of phenolic acids^*a*^^,*b*^

Compound	MW (g/mol)	NHA	NHD	NRB	pKa_1_	TPSA (Å^2^)	Consensus Log *P*_o/w_	Log *S* (ESOL)
Caffeic acid	180.16	4	3	2	4.62	77.76	0.93	−1.89
Chlorogenic acid	354.31	9	6	5	3.5	164.75	−0.39	−1.62
*p*-Coumaric acid	164.16	3	2	2	4.64	57.53	1.26	−2.02
Ferulic acid	194.18	4	2	3	4.58	66.76	1.36	−2.11
Rosmarinic acid	360.31	8	5	7	3.57	144.52	1.58	−3.44
Gallic acid	170.12	5	4	1	4.21	97.99	0.21	−1.64
Salicylic acid	138.12	3	2	1	2.97	57.53	1.13	−2.50
Syringic acid	198.17	5	2	3	4.34	75.99	0.99	−1.84
Vanillic acid	168.15	4	2	2	4.51	66.76	1.08	−2.02

^*a*^ MW—molecular weight; NHA—number of H-bond acceptors; NHD—number of H-bond donors; NRB—number of rotatable bonds; pKa_1_—acid dissociation constant; TPSA—topological polar surface area; Consensus Log *P*_o/w_—partition coefficient between *n*-octanol and water, mean of 5 prediction models; Log *S* (ESOL)—aqueous solubility, ESOL model.

^*b*^ Computed by SwissADME. pKa values obtained from PubChem.

**TABLE 4:** The predicted pharmacokinetic properties of phenolic acids^*a*^^,*b*^

Compound	GIA	BBB	P-gp	CYP1A2 inhibitor	CYP2C19 inhibitor	CYP2C9 inhibitor	CYP2D6 inhibitor	CYP3A4 inhibitor	Log *K*_p_ (cm/s)
Caffeic acid	High	No	No	No	No	No	No	No	−6.58
Chlorogenic acid	Low	No	No	No	No	No	No	No	−8.76
*p*-Coumaric acid	High	Yes	No	No	No	No	No	No	−6.26
Ferulic acid	High	Yes	No	No	No	No	No	No	−6.41
Rosmarinic acid	Low	No	No	No	No	No	No	No	−6.82
Gallic acid	High	No	No	No	No	No	No	Yes	−6.84
Salicylic acid	High	Yes	No	No	No	No	No	No	−5.54
Syringic acid	High	No	No	No	No	No	No	No	−6.77
Vanillic acid	High	No	No	No	No	No	No	No	−6.31

^*a*^ GIA—gastrointestinal absorption; BBB—blood brain barrier permeability; P-gb—P-glycoprotein substrate; CYP—cytochrome; Log *K*_p_—skin permeability coefficient.

^*b*^ Computed by SwissADME.

**TABLE 5:** The toxicity predictions of phenolic acids^*c*^

Compound	Acute oral toxicity (LD50) (mg/kg)^*a*^	Cytotoxicity^*a*^	AMES toxicity^*b*^	hERG I inhibitor^*b*^	hERG II inhibitor^*b*^	Hepato-toxicity^*b*^	Skin sensitization^*b*^
Caffeic acid	2,980	Inactive	No	No	No	No	No
Chlorogenic acid	5,000	Inactive	No	No	No	No	No
*p*-Coumaric acid	2,850	Inactive	No	No	No	No	No
Ferulic acid	1,772	Inactive	No	No	No	No	No
Rosmarinic acid	5,000	Inactive	No	No	No	No	No
Gallic acid	2,000	Inactive	No	No	No	No	No
Salicylic acid	1,034	Inactive	No	No	No	No	No
Syringic acid	1,700	Inactive	No	No	No	No	No
Vanillic acid	2,000	Inactive	No	No	No	No	No

^*a*^ Computed using Protox 3.0.

^*b*^ Computed using pkCSM.

^*c*^ Computed by pkCSM and Protox 3.0.

The drug-likeness of the tested phenolic acids, except chlorogenic acid, complies with Lipinski’s rule of five (molecular weight ≤500 Da, number of H-bond acceptors ≤10, number of H-bond donors ≤5, calculated log*P* ≤ 5) (79). Chlorogenic acid exceeds the limit of H-bond donors. Chlorogenic acid and also rosmarinic acid have the highest topological polar surface area (TPSA) values exceeding 140 Å^2^ (see Table 3). The large TPSA value suggests low membrane permeability and, therefore, poor oral bioavailability (80). It coincides with the predicted low gastrointestinal absorption of chlorogenic and rosmarinic acids (Table 4). The increase in surface polarity negatively correlates with the antibacterial activity (e.g., MIC values), suggesting that highly polar molecules have difficulties penetrating the bacterial cell membrane. *p*-Coumaric acid and salicylic acid, which both have TPSA < 60 Å^2^, have shown arguably the most potent antibacterial activity against both planktonic bacteria and biofilms. Due to the higher lipophilicity, it’s estimated that *p*-coumaric acid, ferulic acid, and salicylic acid could cross the blood-brain barrier (BBB) (Table 4).

Another physicochemical parameter, which can be used to explain the antibacterial potency of these compounds, is pKa (Table 3). All of the tested phenolic acids can be classified as weak acids (pKa 2.97–4.64). Coupled with high TPSA values and larger molecular mass, chlorogenic acid and rosmarinic acid have low pKa values (<3.6). Therefore, during the antibacterial testing assays, the pH > pKa, and most of the molecules are in a dissociated form. Dissociated molecules cannot penetrate the bacterial cell membrane as effectively as undissociated ones. Undissociated phenolic acids can use passive diffusion to cross the cell membrane, followed by the acidification of cytoplasm and denaturation of proteins (55). Therefore, the antibacterial effect of chlorogenic and rosmarinic acids is poorer than that of other phenolic acids, which have higher pKa values. Additionally, the antibiofilm effect of molecules is reliant on the pKa and pH of the media (81).

In case of topical administration against dermatological infections, the skin permeability of compounds is of importance. Salicylic acid would have the highest chance of skin permeability (the least negative Log *K*_p_ value), while chlorogenic acid would have the worst, respectively (Table 4). Overall, the skin permeation ability of phenolic acids can be classified as poor, as the Log *K*_p_ values are overall lower than −6 cm/s. Nevertheless, the skin permeability of these compounds could be improved by encapsulating them in specialized drug delivery systems.

None of the compounds are predicted to be a substrate of P-glycoprotein. Except for gallic acid and CYP3A4 enzyme, it’s predicted that none of the compounds would be inhibitors of the main CYP enzymes (Table 4). This indicates a potentially safer metabolic profile with fewer interactions with other substances. The generated toxicity profile of these compounds is favorable, with no mutagenic (AMES), cardio (hERG I and hERG II), or hepatotoxicity predicted (Table 5). No skin sensitization is anticipated, which is of importance since these compounds were tested against bacterial species characteristic of wound infections and would need to be applied topically. The predicted LD50 values range from 1,034 to 5,000 mg/kg, indicating quite low acute toxicity risk. According to the GHS classification of chemicals, phenolic acids can be classified in acute toxicity hazard category 4 or category 5 (82). Based on the prediction (probability 0.80–0.97), these compounds are not likely to be cytotoxic. Nevertheless, these results were obtained *in silico* and would need to be validated by appropriate *in vitro* and *in vivo* tests in the future. Moreover, if these compounds could be incorporated into novel drug delivery systems like nanoparticles, hydrogels, scaffolds, etc., designed for enhanced dermal delivery combined with controlled drug release properties, the biocompatibility of such systems should be researched in depth.

### Conclusions

Tested phenolic acids show structure-dependent, bactericidal antibacterial activity against bacterial species characteristic of wound infections. The antibacterial activity negatively correlates with the polarity of the molecules. *p*-Coumaric acid and salicylic acid, both free carboxylic acids with only one hydroxyl group on the benzene ring and TPSA < 60 Å^2^, showed the most potent antibacterial activity. Molecules with TPSA > 140 Å^2^ coupled with pKa < 3.6 showed the poorest antibacterial effect, possibly due to the inability of dissociated molecules to cross the bacterial cell membrane. Under the tested conditions, the bacterial strains increased the extracellular environment pH through their metabolism. The change in extracellular pH could be used as an indicator of bacterial viability, since at MBC concentration, the change in pH is close to none, while at sub-MIC concentrations, it reaches that of the controls. Phenolic acids, which have a catechol or pyrogallol group in their structure, exhibit color change under alkaline conditions, likely due to oxidation to quinones followed by reactions with amino compounds and proteins. Gram-positive strain biofilms are more susceptible to phenolic acid treatment. In addition to biofilm biomass reduction, phenolic acids can induce morphological changes in both biofilm and planktonic bacteria. Potential synergic effects with conventional antibiotics, as well as encapsulation in topical drug delivery systems, and the biocompatibility studies of said systems should be explored in the future.

## Data Availability

Data are available within the article and its Supplemental material. Additional data are available upon request from the corresponding authors.

## SUPPLEMENTAL MATERIAL

Graphical abstract spectrum.01336-26-s0001.tiff Graphical abstract.

Supplemental tables spectrum.01336-26-s0002.pdf Tables S1 to S6.

ASM does not own the copyrights to Supplemental Material that may be linked to, or accessed through, an article. The authors have granted ASM a non-exclusive, world-wide license to publish the Supplemental Material files. Please contact the corresponding author directly for reuse.
