# Supplementary material for: Antibacterial and antibiofilm effects of natural phenolic acids: Insights into structure-function relationships, extracellular pH, and morphological changes
Source: Microbiol Spectr. 2026 Jul 17;14(8):e01336-26. doi: 10.1128/spectrum.01336-26 (PMC13436270; doi:10.1128/spectrum.01336-26)
Supplement: Supplementary file 1 [file spectrum.01336-26-s0002.pdf]

<u>**Supplementary Materials**</u>

Antibacterial and antibiofilm effects of natural phenolic acids: insights into structure-function relationships, extracellular pH and morphological changes

Ance Bārzdiņa, Renāte Teterovska, Daniela Paula Prudņikova, Renārs Broks, Krisjanis Smits, Ingus Skadiņš, Dagnija Loca, Aiva Plotniece, Arkadij Sobolev, Karlis Pajuste, Agnese Brangule, and Dace Bandere

Table S1. Stock concentrations and their preparation methods.

Compound	Concentration range of stock solutions (mg/mL)	DMSO concentration in stock	Preparation method
Chlorogenic acid	40-160	10%	The weighed amount of phenolic acid was dispersed in pure DMSO (≥99.9%) using a vortex mixer (TX4 Digital IR Vortex Mixer, VELP Scientifica Srl, Usmate, Italy). Then distilled water was added until the final volume was reached. The dispersion was further mixed using a vortex mixer. If any undissolved drug remained, the solution was placed in a water bath at 50°C until fully dissolved. The solution was kept in a water bath for less than 5 min.
Rosmarinic acid	40-80	10%	
Gallic acid	40-160	10%	
Quinic acid	40-80	10%	
Tannic acid	40-80	10%	
Caffeic acid	40-160	40%	The weighed amount of phenolic acid was fully dissolved in pure DMSO (≥99.9%) using a vortex mixer. Then, distilled water was slowly added dropwise until the final volume was achieved. The solution was carefully mixed. If any precipitate formed, the dispersion was placed in a water bath at 50°C (60°C for syringic acid) until a clear solution was obtained. The solution was kept in a water bath for less than 5 min.
p-Coumaric acid	20-80	40%	
Ferulic acid	40-80	40%	
Salicylic acid	40-80	40%	
Syringic acid	40-80	40%	
Vanillic acid	40-80	40%	

Table S2. MBC/MIC ratio of tested compounds.

Compound	MBC/MIC ratio									
	Gram-positive strains				Gram-negative strains					
	***S. aureus* ATCC 6538**	***S. aureus* ATCC 25923**	***S. epidermidis* ATCC 12228**	***B. subtilis* ATCC 6633**	***E. coli* ATCC 35218**	***E. coli* ATCC 25922**	***P. aeruginosa* ATCC 27853**	***K. quasipneumoniae* ATCC 700603**	***K. pneumoniae* ATCC 10031**	***P. mirabilis* ATCC 43071**
Caffeic acid	1	2	1	2	1	1	1	2	2	2
Chlorogenic acid	1	1	2	1	1	1	1	1	1	1
p-Coumaric acid	2	2	2	4	1	1	1	2	2	1
Ferulic acid	2	1	1	2	2	1	1	2	2	2
Rosmarinic acid	2	2	2	1	1	1	2	2	1	1
Gallic acid	1	1	1	2	2	1	2	1	1	2
Salicylic acid	1	2	1	1	2	2	2	2	2	2
Syringic acid	2	2	2	1	1	2	2	1	1	2
Vanillic acid	2	1	1	1	2	2	2	1	1	2
Quinic acid	1	1	1	1	1	1	2	1	1	1
Tannic acid	32	8	32	-	-	-	-	-	-	-

Table S3. The average pH ± SD before and after 24-hour incubation of the MIC assay. NT-not tested.

**Bacterial strain**	**Compound**	**Concentration**	**pH**	
			**Before incubation**	**After incubation**
***S. aureus* ATCC 6538**	Bacterial control		7.49 ± 0.01	7.58 ± 0.05
	Caffeic acid	2x MIC	4.66 ± 0.03	4.92 ± 0.01
		MIC	5.00 ± 0.00	5.03 ± 0.01
		1/2x MIC	5.79 ± 0.01	5.66 ± 0.03
	Chlorogenic acid	2x MIC	NT	NT
		MIC	4.04 ± 0.02	3.95 ± 0.04
		1/2x MIC	4.68 ± 0.01	6.80 ± 0.27
	p-Coumaric acid	2x MIC	4.96 ± 0.00	5.07 ± 0.02
		MIC	5.64 ± 0.01	5.91 ± 0.02
		1/2x MIC	6.63 ± 0.01	6.69 ± 0.08
	Ferulic acid	2x MIC	5.04 ± 0.01	5.14 ± 0.00
		MIC	5.83 ± 0.01	5.59 ± 0.01
		1/2x MIC	6.75 ± 0.01	7.12 ± 0.06
	Rosmarinic acid	2x MIC	3.86 ± 0.03	3.75 ± 0.01
		MIC	4.63 ± 0.04	4.76 ± 0.04
		1/2x MIC	5.80 ± 0.01	7.20 ± 0.05
	Gallic acid	2x MIC	4.04 ± 0.01	4.16 ± 0.02
		MIC	4.39 ± 0.01	4.53 ± 0.02
		1/2x MIC	4.84 ± 0.01	7.05 ± 0.01
	Salicylic acid	2x MIC	4.27 ± 0.04	4.39 ± 0.03
		MIC	5.09 ± 0.01	5.45 ± 0.01
		1/2x MIC	6.31 ± 0.01	5.91 ± 0.01
	Syringic acid	2x MIC	4.66 ± 0.01	4.69 ± 0.01
		MIC	5.06 ± 0.03	5.21 ± 0.01
		1/2x MIC	5.98 ± 0.04	6.25 ± 0.03
	Vanillic acid	2x MIC	4.91 ± 0.03	5.00 ± 0.01
		MIC	5.75 ± 0.08	5.58 ± 0.01
		1/2x MIC	6.64 ± 0.02	7.45 ± 0.04
	Quinic acid	2x MIC	3.55 ± 0.01	3.53 ± 0.01
		MIC	4.00 ± 0.01	4.08 ± 0.03
		1/2x MIC	4.69 ± 0.01	6.56 ± 0.03

Bacterial strain	Compound	Concentration	pH	
			Before incubation	After incubation
***S. aureus* ATCC 25923**	Bacterial control		7.54 ± 0.01	8.04 ± 0.04
	Caffeic acid	2x MIC	4.66 ± 0.03	4.89 ± 0.01
		MIC	5.00 ± 0.00	6.72 ± 0.22
		1/2x MIC	5.79 ± 0.01	7.81 ± 0.02
	Chlorogenic acid	2x MIC	NT	NT
		MIC	4.04 ± 0.02	4.22 ± 0.01
		1/2x MIC	4.68 ± 0.01	7.65 ± 0.01
	p-Coumaric acid	2x MIC	4.96 ± 0.00	5.29 ± 0.00
		MIC	5.64 ± 0.01	7.48 ± 0.01
		1/2x MIC	6.63 ± 0.01	8.14 ± 0.01
	Ferulic acid	2x MIC	4.70 ± 0.01	4.86 ± 0.01
		MIC	5.04 ± 0.01	5.55 ± 0.07
		1/2x MIC	5.83 ± 0.01	7.59 ± 0.04
	Rosmarinic acid	2x MIC	3.86 ± 0.03	3.89 ± 0.03
		MIC	4.63 ± 0.04	6.60 ± 0.08
		1/2x MIC	5.80 ± 0.01	7.85 ± 0.01
	Gallic acid	2x MIC	NT	NT
		MIC	4.04 ± 0.01	4.26 ± 0.01
		1/2x MIC	4.39 ± 0.01	5.38 ± 0.01
	Salicylic acid	2x MIC	4.27 ± 0.04	4.53 ± 0.01
		MIC	5.09 ± 0.01	6.11 ± 0.08
		1/2x MIC	6.31 ± 0.01	7.51 ± 0.01
	Syringic acid	2x MIC	4.66 ± 0.01	4.87 ± 0.01
		MIC	5.06 ± 0.03	6.60 ± 0.13
		1/2x MIC	5.98 ± 0.04	7.99 ± 0.04
	Vanillic acid	2x MIC	4.54 ± 0.01	4.68 ± 0.01
		MIC	4.91 ± 0.03	5.28 ± 0.06
		1/2x MIC	5.75 ± 0.08	7.70 ± 0.11
	Quinic acid	2x MIC	3.55 ± 0.01	3.58 ± 0.04
		MIC	4.00 ± 0.01	4.40 ± 0.04
		1/2x MIC	4.69 ± 0.01	7.69 ± 0.06

Bacterial strain	Compound	Concentration	pH	
			Before incubation	After incubation
***S. epidermidis* ATCC 12228**	Bacterial control		7.46 ± 0.01	7.54 ± 0.04
	Caffeic acid	2x MIC	4.66 ± 0.03	4.71 ± 0.04
		MIC	5.00 ± 0.00	5.14 ± 0.02
		1/2x MIC	5.79 ± 0.01	6.71 ± 0.01
	Chlorogenic acid	2x MIC	4.04 ± 0.02	3.99 ± 0.01
		MIC	4.68 ± 0.01	6.13 ± 0.01
		1/2x MIC	5.80 ± 0.06	6.74 ± 0.02
	p-Coumaric acid	2x MIC	4.96 ± 0.00	5.07 ± 0.03
		MIC	5.64 ± 0.01	5.98 ± 0.01
		1/2x MIC	6.63 ± 0.01	7.07 ± 0.01
	Ferulic acid	2x MIC	4.70 ± 0.01	4.85 ± 0.01
		MIC	5.04 ± 0.01	5.22 ± 0.02
		1/2x MIC	5.83 ± 0.01	6.55 ± 0.01
	Rosmarinic acid	2x MIC	3.86 ± 0.03	3.79 ± 0.04
		MIC	4.63 ± 0.04	4.74 ± 0.07
		1/2x MIC	5.80 ± 0.01	6.59 ± 0.02
	Gallic acid	2x MIC	4.04 ± 0.01	4.19 ± 0.02
		MIC	4.39 ± 0.01	4.50 ± 0.01
		1/2x MIC	4.84 ± 0.01	6.24 ± 0.07
	Salicylic acid	2x MIC	4.27 ± 0.04	4.29 ± 0.01
		MIC	5.09 ± 0.01	5.41 ± 0.09
		1/2x MIC	6.31 ± 0.01	6.59 ± 0.01
	Syringic acid	2x MIC	4.66 ± 0.01	4.71 ± 0.01
		MIC	5.06 ± 0.03	5.21 ± 0.01
		1/2x MIC	5.98 ± 0.04	6.53 ± 0.03
	Vanillic acid	2x MIC	4.54 ± 0.01	4.60 ± 0.01
		MIC	4.91 ± 0.03	5.03 ± 0.02
		1/2x MIC	5.75 ± 0.08	6.28 ± 0.01
	Quinic acid	2x MIC	3.55 ± 0.01	3.61 ± 0.04
		MIC	4.00 ± 0.01	4.11 ± 0.03
		1/2x MIC	4.69 ± 0.01	6.31 ± 0.01

Bacterial strain	Compound	Concentration	pH	
			Before incubation	After incubation
B. subtilis ATCC 6633	Bacterial control		7.47 ± 0.01	8.82 ± 0.01
	Caffeic acid	2x MIC	4.66 ± 0.03	5.03 ± 0.13
		MIC	5.00 ± 0.00	7.69 ± 0.18
		1/2x MIC	5.79 ± 0.01	8.38 ± 0.02
	Chlorogenic acid	2x MIC	4.04 ± 0.02	4.20 ± 0.01
		MIC	4.68 ± 0.01	7.38 ± 0.06
		1/2x MIC	5.80 ± 0.06	8.53 ± 0.01
	p-Coumaric acid	2x MIC	5.64 ± 0.01	7.74 ± 0.03
		MIC	6.63 ± 0.01	8.42 ± 0.01
		1/2x MIC	7.02 ± 0.01	8.73 ± 0.03
	Ferulic acid	2x MIC	5.04 ± 0.01	5.52 ± 0.05
		MIC	5.83 ± 0.01	8.07 ± 0.05
		1/2x MIC	6.75 ± 0.01	8.50 ± 0.02
	Rosmarinic acid	2x MIC	3.86 ± 0.03	4.16 ± 0.02
		MIC	4.63 ± 0.04	7.39 ± 0.07
		1/2x MIC	5.80 ± 0.01	8.49 ± 0.04
	Gallic acid	2x MIC	4.39 ± 0.01	4.65 ± 0.04
		MIC	4.84 ± 0.01	7.36 ± 0.10
		1/2x MIC	5.62 ± 0.05	8.61 ± 0.03
	Salicylic acid	2x MIC	4.27 ± 0.04	4.72 ± 0.01
		MIC	5.09 ± 0.01	7.94 ± 0.08
		1/2x MIC	6.31 ± 0.01	8.60 ± 0.02
	Syringic acid	2x MIC	4.66 ± 0.01	4.93 ± 0.01
		MIC	5.06 ± 0.03	7.75 ± 0.03
		1/2x MIC	5.98 ± 0.04	8.70 ± 0.03
	Vanillic acid	2x MIC	4.54 ± 0.01	4.99 ± 0.04
		MIC	4.91 ± 0.03	7.90 ± 0.04
		1/2x MIC	5.75 ± 0.08	8.76 ± 0.03
	Quinic acid	2x MIC	4.00 ± 0.01	4.18 ± 0.01
		MIC	4.69 ± 0.01	7.42 ± 0.04
		1/2x MIC	5.81 ± 0.03	8.62 ± 0.02

Bacterial strain	Compound	Concentration	pH	
			**Before incubation**	**After incubation**
***E. coli* ATCC 35218**	Bacterial control		7.56 ± 0.01	8.83 ± 0.03
	Caffeic acid	2x MIC	NT	NT
		MIC	4.66 ± 0.03	5.85 ± 0.42
		1/2x MIC	5.00 ± 0.00	8.39 ± 0.05
	Chlorogenic acid	2x MIC	NT	NT
		MIC	4.04 ± 0.02	5.23 ± 0.01
		1/2x MIC	4.68 ± 0.01	8.44 ± 0.04
	p-Coumaric acid	2x MIC	4.64 ± 0.01	5.04 ± 0.01
		MIC	4.96 ± 0.00	7.68 ± 0.01
		1/2x MIC	5.64 ± 0.01	8.69 ± 0.04
	Ferulic acid	2x MIC	4.70 ± 0.01	5.09 ± 0.01
		MIC	5.04 ± 0.01	7.68 ± 0.04
		1/2x MIC	5.83 ± 0.01	8.69 ± 0.02
	Rosmarinic acid	2x MIC	NT	NT
		MIC	3.86 ± 0.03	5.20 ± 0.08
		1/2x MIC	4.63 ± 0.04	8.38 ± 0.01
	Gallic acid	2x MIC	4.04 ± 0.01	4.28 ± 0.03
		MIC	4.39 ± 0.01	5.06 ± 0.06
		1/2x MIC	4.84 ± 0.01	8.43 ± 0.04
	Salicylic acid	2x MIC	4.27 ± 0.04	5.17 ± 0.06
		MIC	5.09 ± 0.01	7.78 ± 0.10
		1/2x MIC	6.31 ± 0.01	8.69 ± 0.10
	Syringic acid	2x MIC	NT	NT
		MIC	4.66 ± 0.01	5.17 ± 0.06
		1/2x MIC	5.06 ± 0.03	8.24 ± 0.02
	Vanillic acid	2x MIC	4.54 ± 0.01	4.89 ± 0.02
		MIC	4.91 ± 0.03	7.78 ± 0.01
		1/2x MIC	5.75 ± 0.08	8.70 ± 0.03
	Quinic acid	2x MIC	3.55 ± 0.01	3.68 ± 0.03
		MIC	4.00 ± 0.01	4.73 ± 0.24
		1/2x MIC	4.69 ± 0.01	8.50 ± 0.03

Bacterial strain	Compound	Concentration	pH	
			Before incubation	After incubation
***E. coli* ATCC 25922**	Bacterial control		7.47 ± 0.01	8.81 ± 0.02
	Caffeic acid	2x MIC	4.66 ± 0.03	5.06 ± 0.07
		MIC	5.00 ± 0.00	7.71 ± 0.01
		1/2x MIC	5.79 ± 0.01	8.65 ± 0.01
	Chlorogenic acid	2x MIC	NT	NT
		MIC	4.04 ± 0.02	4.46 ± 0.01
		1/2x MIC	4.68 ± 0.01	8.21 ± 0.04
	p-Coumaric acid	2x MIC	4.64 ± 0.01	4.81 ± 0.01
		MIC	4.96 ± 0.00	5.95 ± 0.04
		1/2x MIC	5.64 ± 0.01	8.24 ± 0.02
	Ferulic acid	2x MIC	4.70 ± 0.01	4.94 ± 0.02
		MIC	5.04 ± 0.01	6.35 ± 0.08
		1/2x MIC	5.83 ± 0.01	8.38 ± 0.06
	Rosmarinic acid	2x MIC	3.86 ± 0.03	4.16 ± 0.02
		MIC	4.63 ± 0.04	7.45 ± 0.04
		1/2x MIC	5.80 ± 0.01	8.53 ± 0.04
	Gallic acid	2x MIC	4.04 ± 0.01	4.29 ± 0.01
		MIC	4.39 ± 0.01	4.92 ± 0.01
		1/2x MIC	4.84 ± 0.01	8.16 ± 0.05
	Salicylic acid	2x MIC	4.27 ± 0.04	4.64 ± 0.07
		MIC	5.09 ± 0.01	7.60 ± 0.01
		1/2x MIC	6.31 ± 0.01	8.36 ± 0.02
	Syringic acid	2x MIC	4.66 ± 0.01	4.87 ± 0.01
		MIC	5.06 ± 0.03	7.55 ± 0.10
		1/2x MIC	5.98 ± 0.04	8.60 ± 0.01
	Vanillic acid	2x MIC	4.54 ± 0.01	4.74 ± 0.02
		MIC	4.91 ± 0.03	7.63 ± 0.04
		1/2x MIC	5.75 ± 0.08	8.52 ± 0.01
	Quinic acid	2x MIC	3.55 ± 0.01	3.65 ± 0.03
		MIC	4.00 ± 0.01	4.34 ± 0.05
		1/2x MIC	4.69 ± 0.01	8.05 ± 0.01

Bacterial strain	Compound	Concentration	pH	
			**Before incubation**	**After incubation**
***P. aeruginosa* ATCC 27853**	Bacterial control		7.48 ± 0.01	8.76 ± 0.01
	Caffeic acid	2x MIC	4.66 ± 0.03	6.50 ± 0.66
		MIC	5.00 ± 0.00	7.94 ± 0.04
		1/2x MIC	5.79 ± 0.01	8.67 ± 0.04
	Chlorogenic acid	2x MIC	NT	NT
		MIC	4.04 ± 0.02	5.45 ± 0.21
		1/2x MIC	4.68 ± 0.01	8.26 ± 0.05
	p-Coumaric acid	2x MIC	4.64 ± 0.01	5.32 ± 0.03
		MIC	4.96 ± 0.00	7.93 ± 0.01
		1/2x MIC	5.64 ± 0.01	8.62 ± 0.03
	Ferulic acid	2x MIC	NT	NT
		MIC	4.70 ± 0.01	5.74 ± 0.01
		1/2x MIC	5.04 ± 0.01	8.39 ± 0.02
	Rosmarinic acid	2x MIC	3.86 ± 0.03	4.33 ± 0.06
		MIC	4.63 ± 0.04	7.50 ± 0.02
		1/2x MIC	5.80 ± 0.01	8.49 ± 0.03
	Gallic acid	2x MIC	4.39 ± 0.01	4.70 ± 0.04
		MIC	4.84 ± 0.01	7.45 ± 0.06
		1/2x MIC	5.62 ± 0.05	8.61 ± 0.03
	Salicylic acid	2x MIC	4.27 ± 0.04	4.71 ± 0.15
		MIC	5.09 ± 0.01	7.72 ± 0.08
		1/2x MIC	6.31 ± 0.01	8.59 ± 0.04
	Syringic acid	2x MIC	4.66 ± 0.01	5.15 ± 0.04
		MIC	5.06 ± 0.03	8.07 ± 0.13
		1/2x MIC	5.98 ± 0.04	8.68 ± 0.02
	Vanillic acid	2x MIC	4.54 ± 0.01	4.84 ± 0.11
		MIC	4.91 ± 0.03	7.78 ± 0.01
		1/2x MIC	5.75 ± 0.08	8.67 ± 0.01
	Quinic acid	2x MIC	4.00 ± 0.01	4.45 ± 0.13
		MIC	4.69 ± 0.01	7.81 ± 0.18
		1/2x MIC	5.81 ± 0.03	8.53 ± 0.00

Bacterial strain	Compound	Concentration	pH	
			Before incubation	After incubation
K. pneumoniae ATCC 10031	Bacterial control		7.59 ± 0.01	8.10 ± 0.01
	Caffeic acid	2x MIC	4.66 ± 0.03	4.90 ± 0.07
		MIC	5.00 ± 0.00	7.54 ± 0.01
		1/2x MIC	5.79 ± 0.01	7.71 ± 0.02
	Chlorogenic acid	2x MIC	NT	NT
		MIC	4.04 ± 0.02	4.08 ± 0.01
		1/2x MIC	4.68 ± 0.01	7.55 ± 0.10
	p-Coumaric acid	2x MIC	4.96 ± 0.00	5.35 ± 0.04
		MIC	5.64 ± 0.01	7.46 ± 0.06
		1/2x MIC	6.63 ± 0.01	7.44 ± 0.01
	Ferulic acid	2x MIC	4.70 ± 0.01	4.87 ± 0.01
		MIC	5.04 ± 0.01	7.52 ± 0.02
		1/2x MIC	5.83 ± 0.01	7.55 ± 0.01
	Rosmarinic acid	2x MIC	NT	NT
		MIC	3.86 ± 0.03	3.91 ± 0.01
		1/2x MIC	4.63 ± 0.04	7.69 ± 0.03
	Gallic acid	2x MIC	4.04 ± 0.01	4.16 ± 0.01
		MIC	4.39 ± 0.01	4.54 ± 0.02
		1/2x MIC	4.84 ± 0.01	7.64 ± 0.01
	Salicylic acid	2x MIC	4.27 ± 0.04	4.45 ± 0.06
		MIC	5.09 ± 0.01	6.34 ± 0.22
		1/2x MIC	6.31 ± 0.01	7.88 ± 0.02
	Syringic acid	2x MIC	NT	NT
		MIC	4.66 ± 0.01	4.70 ± 0.01
		1/2x MIC	5.06 ± 0.03	7.80 ± 0.01
	Vanillic acid	2x MIC	4.54 ± 0.01	4.61 ± 0.01
		MIC	4.91 ± 0.03	5.67 ± 0.06
		1/2x MIC	5.75 ± 0.08	8.01 ± 0.04
	Quinic acid	2x MIC	3.55 ± 0.01	3.55 ± 0.01
		MIC	4.00 ± 0.01	4.05 ± 0.06
		1/2x MIC	4.69 ± 0.01	7.55 ± 0.06

**Bacterial strain**	**Compound**	**Concentration**	**pH**	
			**Before incubation**	**After incubation**
***K. quasipneumoniae* ATCC 700603**	Bacterial control		7.55 ± 0.01	8.44 ± 0.06
	Caffeic acid	2x MIC	4.66 ± 0.03	4.84 ± 0.04
		MIC	5.00 ± 0.00	7.54 ± 0.06
		1/2x MIC	5.79 ± 0.01	7.80 ± 0.12
	Chlorogenic acid	2x MIC	NT	NT
		MIC	4.04 ± 0.02	4.09 ± 0.01
		1/2x MIC	4.68 ± 0.01	7.76 ± 0.04
	p-Coumaric acid	2x MIC	4.96 ± 0.00	5.33 ± 0.01
		MIC	5.64 ± 0.01	7.53 ± 0.02
		1/2x MIC	6.63 ± 0.01	7.48 ± 0.01
	Ferulic acid	2x MIC	4.70 ± 0.01	4.85 ± 0.01
		MIC	5.04 ± 0.01	7.48 ± 0.06
		1/2x MIC	5.83 ± 0.01	7.55 ± 0.01
	Rosmarinic acid	2x MIC	3.86 ± 0.03	3.87 ± 0.01
		MIC	4.63 ± 0.04	7.58 ± 0.02
		1/2x MIC	5.80 ± 0.01	7.97 ± 0.02
	Gallic acid	2x MIC	4.04 ± 0.01	4.32 ± 0.02
		MIC	4.39 ± 0.01	4.61 ± 0.01
		1/2x MIC	4.84 ± 0.01	7.55 ± 0.09
	Salicylic acid	2x MIC	4.27 ± 0.04	4.27 ± 0.06
		MIC	5.09 ± 0.01	7.41 ± 0.05
		1/2x MIC	6.31 ± 0.01	7.82 ± 0.05
	Syringic acid	2x MIC	NT	NT
		MIC	4.66 ± 0.01	4.81 ± 0.02
		1/2x MIC	5.06 ± 0.03	7.73 ± 0.05
	Vanillic acid	2x MIC	4.54 ± 0.01	4.65 ± 0.01
		MIC	4.91 ± 0.03	5.46 ± 0.02
		1/2x MIC	5.75 ± 0.08	7.99 ± 0.01
	Quinic acid	2x MIC	3.55 ± 0.01	3.60 ± 0.01
		MIC	4.00 ± 0.01	4.15 ± 0.01
		1/2x MIC	4.69 ± 0.01	7.83 ± 0.06

Bacterial strain	Compound	Concentration	pH	
			Before incubation	After incubation
***P. mirabilis* ATCC 43071**	Bacterial control		7.48 ± 0.01	8.84 ± 0.03
	Caffeic acid	2x MIC	4.66 ± 0.03	7.27 ± 0.56
		MIC	5.00 ± 0.00	7.88 ± 0.01
		1/2x MIC	5.79 ± 0.01	8.73 ± 0.01
	Chlorogenic acid	2x MIC	NT	NT
		MIC	4.04 ± 0.02	6.02 ± 0.01
		1/2x MIC	4.68 ± 0.01	8.32 ± 0.07
	p-Coumaric acid	2x MIC	4.64 ± 0.01	5.11 ± 0.01
		MIC	4.96 ± 0.00	7.82 ± 0.01
		1/2x MIC	5.64 ± 0.01	8.48 ± 0.04
	Ferulic acid	2x MIC	4.70 ± 0.01	5.07 ± 0.02
		MIC	5.04 ± 0.01	7.81 ± 0.06
		1/2x MIC	5.83 ± 0.01	8.56 ± 0.01
	Rosmarinic acid	2x MIC	3.86 ± 0.03	4.58 ± 0.23
		MIC	4.63 ± 0.04	7.66 ± 0.00
		1/2x MIC	5.80 ± 0.01	8.55 ± 0.01
	Gallic acid	2x MIC	4.04 ± 0.01	4.16 ± 0.02
		MIC	4.39 ± 0.01	4.90 ± 0.02
		1/2x MIC	4.84 ± 0.01	8.07 ± 0.02
	Salicylic acid	2x MIC	4.27 ± 0.04	4.41 ± 0.01
		MIC	5.09 ± 0.01	7.70 ± 0.02
		1/2x MIC	6.31 ± 0.01	8.29 ± 0.01
	Syringic acid	2x MIC	4.66 ± 0.01	4.87 ± 0.06
		MIC	5.06 ± 0.03	7.66 ± 0.03
		1/2x MIC	5.98 ± 0.04	8.76 ± 0.07
	Vanillic acid	2x MIC	4.54 ± 0.01	4.65 ± 0.02
		MIC	4.91 ± 0.03	7.43 ± 0.11
		1/2x MIC	5.75 ± 0.08	8.60 ± 0.04
	Quinic acid	2x MIC	3.55 ± 0.01	3.75 ± 0.02
		MIC	4.00 ± 0.01	4.99 ± 0.47
		1/2x MIC	4.69 ± 0.01	8.30 ± 0.01

Table S4. The photographs of wells after the MIC assay. Abbreviations: MIC – minimum inhibitory concentration, NT- not tested.

	S. aureus **ATCC 6538**	***S. aureus*** **ATCC 25923**	***S. epidermidis*** **ATCC 12228**	***B. subtilis*** **ATCC 6633**	***E. coli*** **ATCC 35218**	***E. coli*** **ATCC 25922**	***K. pneumoniae*** **ATCC 10031**	***K. quasipneumoniae*** **ATCC 700603**	***P. aeruginosa*** **ATCC 27853**	***P. mirabilis*** **ATCC 43071**
**Caffeic acid**										
2×MIC	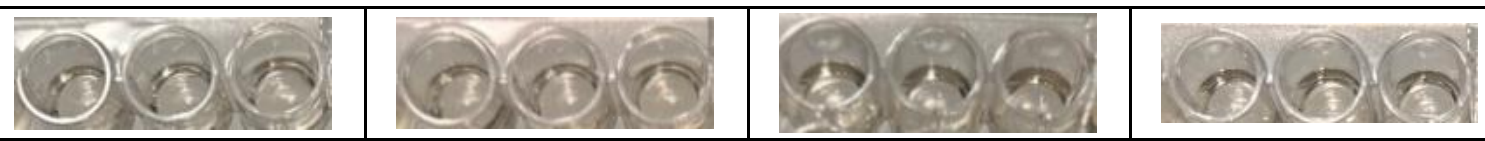				NT	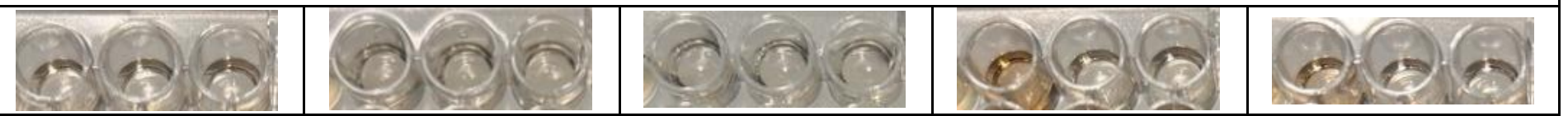				
MIC	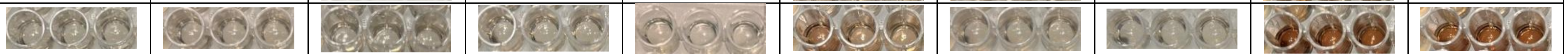									
1/2×MIC	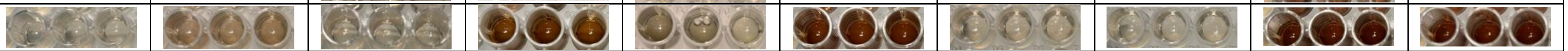									
1/4×MIC	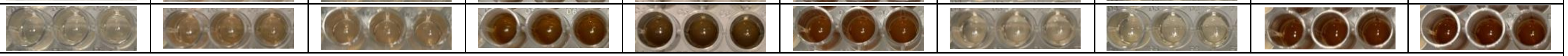									
**Chlorogenic acid**										
2×MIC	NT	NT	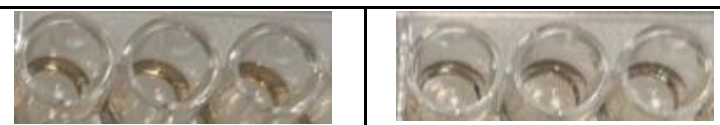		NT	NT	NT	NT	NT	NT
MIC	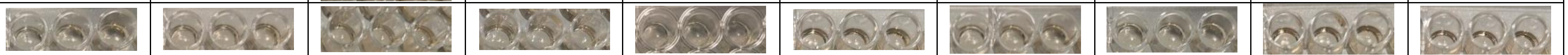									
1/2×MIC	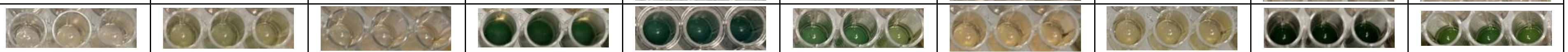									
1/4×MIC	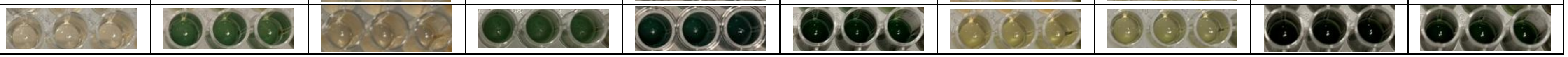									

	S. aureus ATCC 6538	*S. aureus* ATCC 25923	*S. epidermidis* ATCC 12228	*B. subtilis* ATCC 6633	*E. coli* ATCC 35218	*E. coli* ATCC 25922	*K. pneumoniae* ATCC 10031	*K. quasipneumoniae* ATCC 700603	*P. aeruginosa* ATCC 27853	*P. mirabilis* ATCC 43071
**Rosmarinic acid**										
2×MIC					NT		NT			
MIC										
1/2×MIC										
1/4×MIC										
**Gallic acid**										
2×MIC		NT								
MIC										
1/2×MIC										
1/4×MIC										

Table S5. The biofilm prevention concentration (BPC) values of tested compounds (mg/mL).

Compound	BPC (mg/mL)						
	Gram-positive strains		Gram-negative strains				
	S. aureus ATCC 6538	*S. aureus ATCC 25923*	*E. coli ATCC 35218*	*E. coli ATCC 25922*	*P. aeruginosa ATCC 27853*	*K. quasipneumoniae ATCC 700603*	*K. pneumoniae ATCC 10031*
Caffeic acid	2.5	2.5	5.0	2.5	2.5	2.5	5.0
p-Coumaric acid	2.5	0.625	1.25	5.0	2.5	2.5	5.0
Ferulic acid	2.5	1.25	2.5	1.25	10.0	2.5	10.0
Gallic acid	5.0	5.0	5.0	5.0	2.5	5.0	5.0
Salicylic acid	2.5	0.625	2.5	1.25	2.5	2.5	1.25
Syringic acid	2.5	1.25	2.5	2.5	10.0	2.5	2.5
Vanillic acid	2.5	1.25	2.5	2.5	2.5	2.5	2.5
Quinic acid	5.0	2.5	5.0	5.0	1.25	5.0	5.0

Table S6. The biofilm tolerance factor-prevention (BTF-P) values of tested compounds.

Compound	BTF-P						
	Gram-positive strains		Gram-negative strains				
	S. aureus ATCC 6538	***S. aureus ATCC 25923***	***E. coli ATCC 35218***	***E. coli ATCC 25922***	***P. aeruginosa ATCC 27853***	***K. quasipneumoniae ATCC 700603***	***K. pneumoniae ATCC 10031***
Caffeic acid	1	1	1	1	1	1	2
p-Coumaric acid	2	0.5	0.5	2	1	2	4
Ferulic acid	2	0.5	1	0.5	2	1	4
Gallic acid	1	0.5	1	1	1	1	1
Salicylic acid	2	0.5	2	1	2	2	1
Syringic acid	1	0.5	0.5	1	4	0.5	0.5
Vanillic acid	2	0.5	1	1	1	1	1
Quinic acid	1	0.5	1	1	0.5	1	1
